# Mono-, Bis-, and Tris-Chelate Zn(II) Complexes with Imidazo[1,5-*a*]pyridine: Luminescence and Structural Dependence

**DOI:** 10.3390/molecules28093703

**Published:** 2023-04-25

**Authors:** Valerio Cerrato, Giorgio Volpi, Emanuele Priola, Alessia Giordana, Claudio Garino, Roberto Rabezzana, Eliano Diana

**Affiliations:** Department of Chemistry, University of Turin, Via Pietro Giuria 7, 10125 Torino, Italy

**Keywords:** imidazo[1,5-*a*]pyridine, luminescence, fluorescence, zinc complex, quantum yield, Stokes shift

## Abstract

New mono-, bis-, and tris-chelate Zn(II) complexes have been synthesized starting from different Zn(II) salts and employing a fluorescent 1,3-substituted-imidazo[1,5-*a*]pyridine as a chelating ligand. The products have been characterized by single-crystal X-ray diffraction; mass spectrometry; and vibrational spectroscopy. The optical properties have been investigated to compare the performances of mono-, bis-, and tris-chelate forms. The collected data (in the solid state and in solution) elucidate an important modification of the ligand conformation upon metal coordination; which is responsible for a notable increase in the optical performance. An intense modification of the emission quantum yield along the series in the solid state is observed comparing mono-, bis-, and tris-chelate adducts; independently from the anionic ligand introduced by ionic exchange.

## 1. Introduction

The design of luminescent metal complexes for materials science is of fundamental importance in expanding the technological applications of inorganic complexes. In particular, the demand for optically efficient materials prompts researchers to develop new fluorescent organic molecules/ligands and corresponding luminescent complexes suitable for imaging, lightening, and energy-conversion applications [1,2,3,4,5,6,7,8,9]. Their design is driven by the necessity to optimize the optical performance controlling the chemical structure, the chemical stability, and the excited states of the synthesized molecular scaffolds. In this regard, the applications of luminescent complexes of earth-abundant metals such as iron, copper, and zinc are relatively sparse in comparison to the most studied precious transition metals (such as Pt, Ir, Ru, or Re) [10,11,12,13,14,15,16].

In the literature, the investigation of luminescent Zn(II) complexes is well-documented in developing luminescent materials for organic light-emitting diodes (OLEDs) and light-emitting electrochemical cells (LECs) [17,18,19,20,21].

**Scheme 1:**
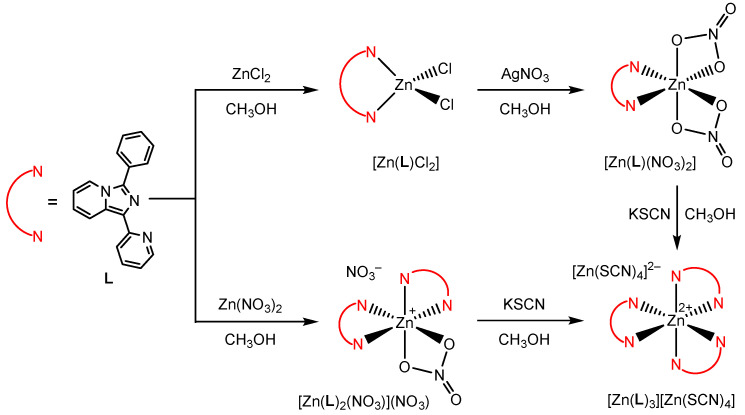
Structures of 3-phenyl-1-(pyridin-2-yl)imidazo[1,5-*a*]pyridine (**L**) and the corresponding mono-, bis-, and tris-chelate Zn(II) complexes.

Here, we describe the synthesis and characterization of a series of Zn(II) complexes, based on the combination of a chelating 1,3-substituted-imidazo[1,5-*a*]pyridine (namely 3-phenyl-1-(pyridin-2-yl)imidazo[1,5-*a*]pyridine **L**, Scheme 1) with three anions (Cl^−^, NO_3_^−^, SCN^–^), acting as ligands or counterions. Our results demonstrate how finely balanced the factors influencing mono-, bis-, or tris-chelation can be and should aid the future rational development of stable and low-cost emitters.

Imidazo[1,5-*a*]pyridine derivatives are well known in the literature due to their intense emission, optical tunability, and ligand capability [22,23,24,25,26,27]. In general, imidazo[1,5-*a*]pyridines have been employed for different applications such as cancer therapy [28,29], confocal microscopy [30,31], down-shifting conversion [32,33], and as molecular sensors [34,35,36,37,38,39,40,41]. When an appropriate substituent (such as OH) is introduced in position three, the modified imidazo[1,5-*a*]pyridine nucleus can be used as an N^O chelating ligand. Indeed, the literature reports several examples of emissive complexes obtained with Zn, Ag, Ni, Co, Pd, and Cu and N^O chelating and emissive 3-substituted imidazo[1,5-*a*]pyridines [11,42,43,44]. In this context, the imidazo[1,5-*a*]pyridine skeleton with a pendant OH-phenyl in position three could be considered equivalent to the well-known 8-hydroxyquinoline in the preparation of widely employed luminescent complexes such as tris-(8-hydroxyquinoline)aluminium, a well-known component of organic light-emitting diodes (OLEDs) [45]. However, if properly modified, this interesting class of heterocyclic compounds could be as versatile as the important families of bipyridine or phenanthroline ligands. As a matter of fact, the insertion of a pendant pyridine (in position one on the imidazo[1,5-*a*]pyridine nucleus) guarantees an N^N bidentate ligand motif, a molecular skeleton suitable for complexation reactions with different transition metal ions [8,46,47,48,49,50,51].

In the present work, 3-phenyl-1-(pyridin-2-yl)imidazo[1,5-*a*]pyridine (**L**) is employed as a ligand to obtain differently structured Zn(II) complexes, leading towards optical modification and quantum-yield increases for promising optical and technological applications. In particular, employing this well-known fluorescent molecule, we obtained four new Zn(II) complexes; two mono-chelate, one bis-chelate, and one tris-chelate derivative. Different ancillary anionic ligands have been evaluated (Cl^−^, NO_3_^−^, SCN^−^) starting from the corresponding Zn(II) salts or by means of ion exchange. Finally, the optical properties have been investigated in order to evaluate the luminescence and the structural dependence, elucidating the importance of ligands’ and counterions’ stoichiometry for the optical performance.

## 2. Results and Discussion

### 2.1. Synthesis

The ligand **L** was prepared as previously reported, by a direct one-pot cyclization synthetic approach, condensing di(2-pyridyl)ketone with benzaldehyde in acetic acid and ammonium acetate [46,47,48,49]. The purity was assessed by TLC, ^1^H, and ^13^C NMR spectroscopy (see Supplementary Materials).

The mono-chelate [Zn(**L**)Cl_2_] complex was synthesized by the reaction of ZnCl_2_ with a stoichiometric amount of **L** in methanol. Successively, [Zn(**L**)(NO_3_)_2_] was obtained by ionic exchange, adding AgNO_3_ to a methanolic solution of [Zn(**L**)Cl_2_]. Afterwards, [Zn(**L**)_3_][Zn(SCN)_4_] was obtained by precipitation, adding KSCN to a solution of [Zn(**L**)(NO_3_)_2_] (see Scheme 1). The bis-chelate [Zn(**L**)_2_(NO_3_)](NO_3_) complex was prepared by reacting Zn(NO_3_)_2_ with a stoichiometric amount of **L** in methanol. Successively, the tris-chelate [Zn(**L**)_3_][Zn(SCN)_4_] was obtained by precipitation, adding KSCN to a solution of [Zn(**L**)_2_(NO_3_)](NO_3_) (see Scheme 1).

All the complexes are yellow crystalline powders and were characterized by mass spectrometry; IR, Raman, and UV-Vis absorption and emission spectroscopies; and single-crystal X-ray diffraction (SC-XRD). Due to the extremely low solubility of these complexes in the common deuterated solvents, it was not possible to perform NMR characterization.

It is worth noting that while the direct reaction of ZnCl_2_ with **L** gives the corresponding mono-chelate [Zn(**L**)Cl_2_] complex, the direct reaction of Zn(NO_3_)_2_ with **L** gives the corresponding bis-chelate [Zn(**L**)_2_(NO_3_)](NO_3_) complex. Thus, we designed a synthetic strategy to obtain the mono-chelate complex with nitrate anions, not obtainable from Zn(NO_3_)_2_ by direct reaction. The adopted synthetic approach employs AgNO_3_ to eliminate chloride anions as an AgCl precipitate.

[Zn(**L**)_3_][Zn(SCN)_4_] was obtained by addition of KSCN to either a solution of mono-chelate [Zn(**L**)(NO_3_)_2_] or a solution of bis-chelate [Zn(**L**)_2_(NO_3_)](NO_3_) precursors (see Scheme 1).

### 2.2. Optical Characterization

**Figure 1:**
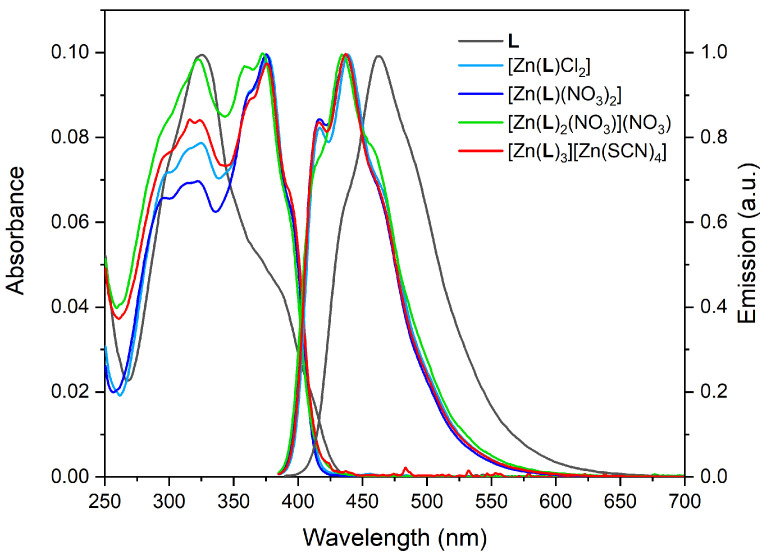
Absorption and normalized emission spectra of **L** and of the corresponding mono-, bis-, and tris-chelate Zn(II) complexes.

The optical data obtained for the free ligand **L** and for the mono-, bis-, and tris-chelate Zn(II) complexes in dichloromethane solution are collected in Table 1, while the absorption and emission spectra are presented in Figure 1.

The free ligand **L** shows its main absorption features in the UV region, in the wavelength range between 280 nm and 380 nm. The main peak is centred at 224 nm, with a shoulder falling at 377 nm and almost no absorption beyond 400 nm.

The corresponding mono-chelate complexes (as well as the bis- and tris-chelate analogues) show three main absorptions: the first at about 310–330 nm, the second in the 350–360 nm range, and the third centred at 370–380 nm with a shoulder at 394–398 nm. All the complexes show a small blue shift when compared to the corresponding free ligand and an intense increase in the low-energy band at about 380 nm.

Imidazo[1,5-*a*]pyridine derivatives are well known in the literature as intense emitters [52,53,54,55,56]. Indeed, 3-phenyl-1-(pyridin-2-yl)imidazo[1,5-*a*]pyridine (**L**) displays an intense fluorescence emission centred at 463 nm (in dichloromethane solution), with a quantum yield of 19% and a remarkable Stokes shift of 79 nm [22,57,58,59,60,61,62].

The emission spectra of all of the obtained complexes in dichloromethane solution show similar profiles and a comparable blue shift (about 20–25 nm) when compared to the corresponding free ligand (see Table 1 and Figure 1). In contrast to what has been reported in the literature for other N^N bidentate ligands, this peculiar observation has been previously reported by us for similar systems [63]. The observed blue shift is not related to the tested ancillary anionic ligand, nor to the tetrahedral or octahedral geometry of the mono-, bis-, and tris-chelate structures. In general, all the collected emissions show a defined and intense structured band centred at about 440 nm (surrounded by two shoulders at 415–420 and 460–465 nm) and more evident than for the free ligand (see Figure 1). This is clear evidence of the well-known intra-ligand π–π∗ character of the electronic transitions responsible for the fluorescence, as expected for a chelating imidazo[1,5-*a*]pyridine coordinated to a closed-shell Zn(II) ion [64,65]. This peculiarity has been previously reported by us for similar mono-chelate Zn(II) complexes as the result of the increased rigidity and the conformational modifications of the ligand (from transoid to cisoid) upon metal coordination [66].

In general, a marked increase in quantum yield can be observed passing from the free ligand to each of the complexes. Comparing mono-, bis-, and tris-chelate complexes in dichloromethane solutions, no appreciable difference was observed in the case of the two nitrate derivatives [(Zn(**L**)(NO_3_)_2_] and [Zn(**L**)_2_(NO_3_)](NO_3_); contrariwise, a notable difference in the quantum yield resulted for the thiocyanate [Zn(**L**)_3_][Zn(SCN)_4_], which showed a lower value (23%). The emission centred at λ = 400–460 nm can be ascribed to an LC (π–π*) electronic transition, in agreement with the vibrational profile and with the assignment reported in the literature for Cu, Ir, and Re analogues [46,47,48,49,51]. In conclusion, negligible differences can be observed in the emission profiles in solution of mono-, bis-, and tris-chelate complexes, even with the different tested anionic ancillary ligands, although the thiocyanate derivative shows a decrease in the optical performance.

**Figure 2:**
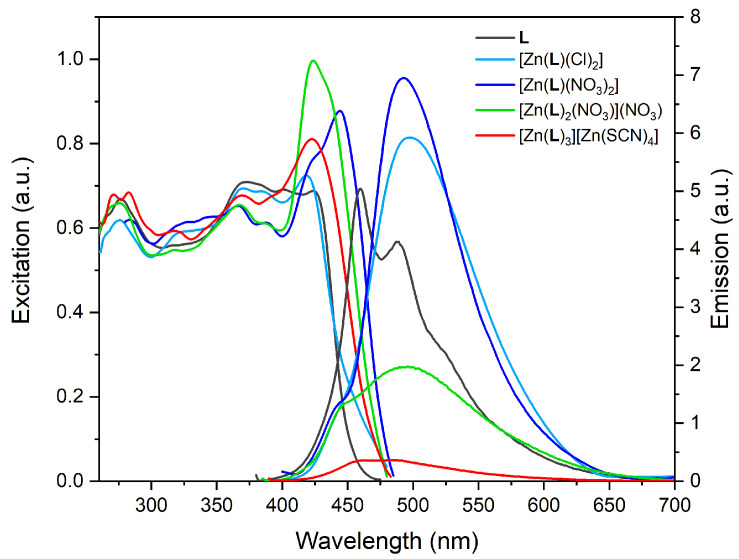
Excitation and emission spectra of **L** and corresponding mono-, bis-, and tris-chelate Zn(II) complexes at the solid state.

The emission spectra of the complexes collected in the solid state (see Table 2 and Figure 2) show notably different profiles and optical performances when compared to the data collected in solution. In general, all the spectra show an emission band around 450–550 nm, with the loss of the vibrational profile. These emissions are red-shifted by about 20–40 nm when compared to the free ligand and by about 40–60 nm with respect to the emissions in solution. Additionally, in this case, an increase in quantum yield can be observed passing from the free ligand (5%) to the mono-chelated complexes (6% and 7% for [Zn(**L**)Cl_2_] and [Zn(**L**)(NO_3_)_2_], respectively). However, the quantum yields dramatically change along the series passing from mono- to bis- and tris-chelate forms (7%, 2%, and <1% respectively), highlighting a decreasing trend as a function of the stoichiometry. Even in the solid state, similar to the behaviour seen in solution, it can be noted that the role of the tested anionic ancillary ligands is almost irrelevant for the optical properties.

### 2.3. Structural Characterization

**Figure 3:**
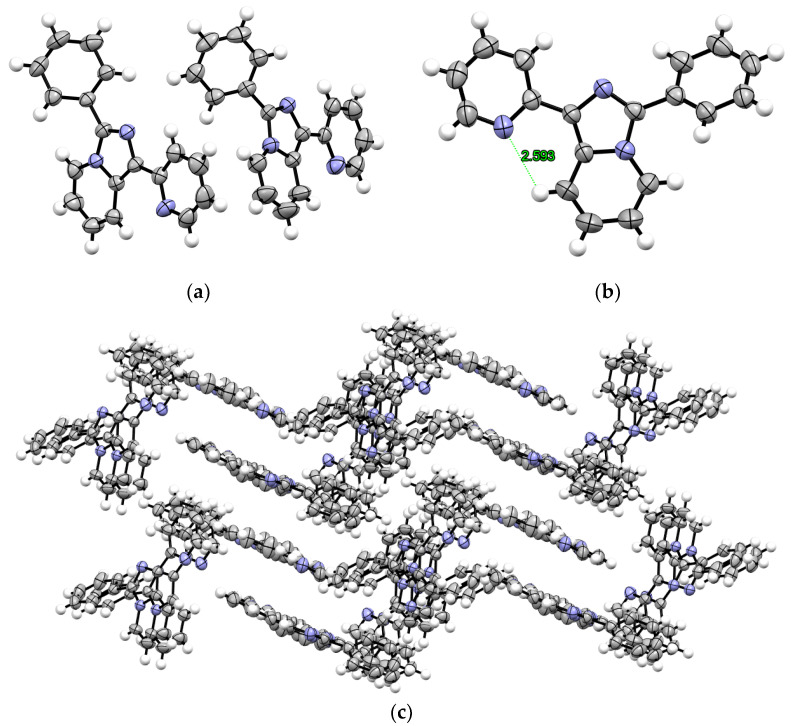
(**a**) Asymmetric unit of **L**; (**b**) intramolecular C–H···N interaction in transoid conformation; (**c**) herringbone motif in crystal packing. Grey: carbon; white: hydrogen; violet: nitrogen–ORTEP plot 50%.

The employed ligand **L** crystallizes from CH_2_Cl_2_ in the *P*-1 space group and its asymmetric unit is formed by two molecules (Figure 3a). This peculiarity (Z > 2) is common for the imidazopyridine family [66]. As can be noticed from the crystal structure, the N atom of the pyridine ring is in a transoid conformation compared to the N atom of the imidazopyridine, and an intramolecular C–H···N bond between the N atom of the pyridine and the proximal H atom of the imidazopyridine can be observed (d(C–H···N) = 2.593(7) Å), Figure 3b). The crystal packing shows the classical herringbone pattern, dominated by slipped π···π stacking and C–H···π contacts (Figure 3c).

The crystal structure of complex [Zn(**L**)Cl_2_] has already been reported and it will not be further discussed [63].

**Figure 4:**
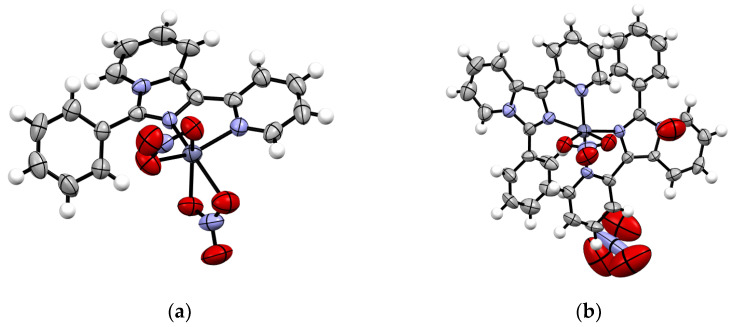
Asymmetric unit of the mono-chelate and bis-chelate complexes: (**a**) [Zn(**L**)(NO_3_)_2_] and (**b**) [Zn(**L**)_2_(NO_3_)](NO_3_). Grey: carbon; white: hydrogen; violet: nitrogen; red: oxygen; violet: zinc–ORTEP plot 50%.

Complex [Zn(**L**)(NO_3_)_2_] crystallizes from methanol in the monoclinic *P*2_1_/*n* space group. The Zn(II) centre coordinates by the chelation of two nitrate ions and one **L** molecule, forming a tetrahedron (Figure 4). The two nitrate ligands are involved in weak C–H···O contacts with the phenyl, pyridyl, and pyridine rings of the imidazopyridine moieties of proximal molecules.

The complex obtained by direct reaction between **L** and Zn(NO_3_)_2_ in ethanol crystallizes as: {[Zn(**L**)_2_(NO_3_)](NO_3_)}·H_2_O with the triclinic centrosymmetric *P*-1 space group (in Figure 4). The asymmetric unit of {[Zn(**L**)_2_(NO_3_)](NO_3_)}·H_2_O consists of one molecule of the complex, where the metal centre is chelated by two **L** molecules and one nitrate, while the second nitrate anion and a water molecule are also included in the crystal packing. The water of crystallization interacts with both coordinated and uncoordinated NO_3_^−^ anions as a donor through strong hydrogen bonds (O–H···O) and with the aromatic moieties as an acceptor through weaker hydrogen bonds (C–H···O). This weak hydrogen interaction (C–H···O) is also observed between the uncoordinated (and disordered) nitrate and the pyridine ring of the ligand.

**Figure 5:**
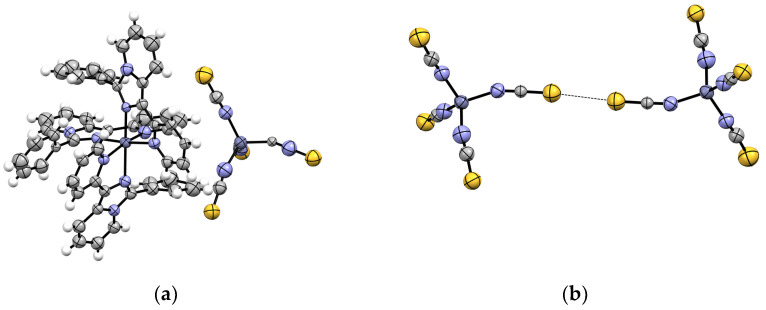
Asymmetric unit (**a**) and anion···anion interaction (**b**) in the crystal structure of complex [Zn(**L**)_3_][Zn(SCN)_4_]. Grey: carbon; white: hydrogen; violet: nitrogen; violet: zinc; yellow: sulphur–ORTEP plot 50%.

The complex [Zn(**L**)_3_][Zn(SCN)_4_], obtained by reaction between KSCN and mono/bis-chelate complexes, crystallizes in the triclinic *P*-1 space group and presents in its asymmetric unit two different ionic fragments: [Zn(**L**)_3_]^2+^ and [Zn(SCN)_4_]^2−^. In the cationic fragment, the Zn(II) ion presents a distorted octahedral environment and, to the best of our knowledge, this is the first example of a tris-chelate complex with imidazo[1,5-*a*]pyridine ligands. Within the molecule, there is an intramolecular interaction between the phenyl and pyridyl rings of different **L**s, and we can suppose that this internal π···π stacking interaction probably contributes to stabilizing the bulky coordination sphere of the Zn(II). The anionic fragment presents a tetrahedral coordination, with all the thiocyanate groups bonded through the N atom and linear (Figure 5a). The crystal packing is dominated by Coulomb forces, but geometry and distance suggest the presence of a chalcogen bond between two thiocyanate groups of proximal anionic fragments (Figure 5b).

**Figure 6:**
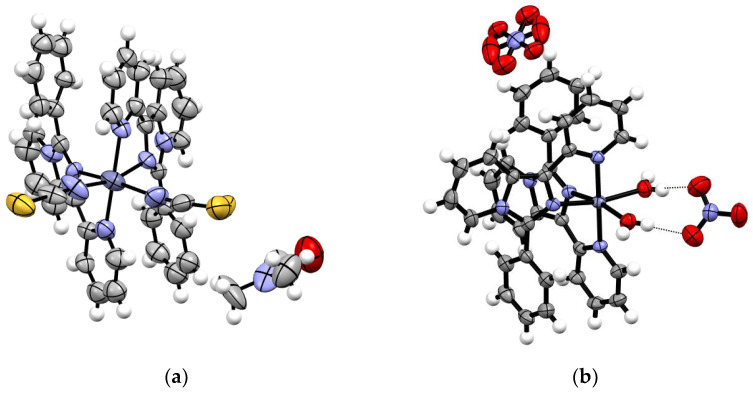
Crystal structure of [Zn(**L**)_2_(SCN)_2_]·DMF (**a**) and [Zn(**L**)_2_(H_2_O)_2_](NO_3_)_2_ (**b**). Grey: carbon; white: hydrogen; violet: nitrogen; red: oxygen; violet: zinc; yellow: sulphur-ORTEP plot 50%.

In addition to the complexes designed for this work, two other products were obtained by crystallization attempts performed in different solvents. The complex [Zn(**L**)_2_(SCN)_2_]·DMF was isolated after redissolution of [Zn(**L**)_3_][Zn(SCN)_4_] in DMF. It crystallizes in the triclinic *P*-1 space group, and the asymmetric unit contains one molecule of the complex and one molecule of solvent (Figure 6). The Zn(II) metal centre coordinates two **L** molecules and two SCN^−^ anions by the N atom in a distorted octahedral geometry. One thiocyanate presents positional disorder. The S atom of the SCN^−^ interacts with a hydrogen atom of the imidazopyridine moiety of another complex molecule.

Similarly, the complex [Zn(**L**)_2_(H_2_O)_2_](NO_3_)_2_ was isolated after redissolution of [Zn(**L**)_2_(NO_3_)](NO_3_) in methanol. It crystallizes in the monoclinic centrosymmetric *C*2/*c* space group and presents in its asymmetric unit one molecule of complex with the Zn(II) ion coordinated by two chelating ligands and two water molecules, forming a regular octahedron (Figure 6); two nitrate anions are present in the structure as counterions. The water molecules interact with a short hydrogen-bond distance with the O atoms of both nitrate anions. Moreover, the aromatic rings of imidazopyridine, pyridine, and phenyl interact via π···π stacking with the aromatic rings of the surrounding molecules.

### 2.4. Vibrational Characterization

**Figure 7:**
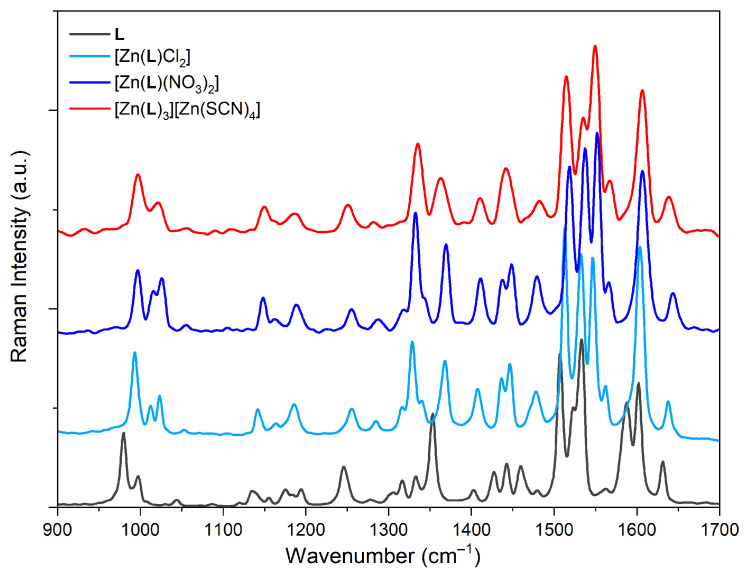
FT-Raman spectra of **L** and of the corresponding mono-chelate [Zn(**L**)(X)_2_] (X = Cl, NO_3_) and tris-chelate complexes.

IR and Raman spectra of all the synthesized complexes have been collected (Table S1, Supplementary Materials). The Raman spectrum of **L** is simple and is used herein for comparison with the spectra of the metal complexes. In Figure 7, we compare the Raman spectra of the different products obtained by the reaction between ZnCl_2_ and **L**, and the successive ion exchanges. The most intense Raman signals of **L** are ring-stretching modes (in the 1450–1600 cm^−1^ spectral region), which are usually slightly shifted to higher wavenumbers by the rigidity imposed by the metal coordination. The pattern of the mono- and tris-chelate complexes is very similar, suggesting that the vibrational modes are not affected by the number of coordinated ligands. This aspect is confirmed by the calculated spectra obtained from a semiempirical tight-binding (TB) method [67,68]. The literature assignment of the **L** signals, based on the DFT method [66], indicates that the signals around 1600 cm^−1^ are mainly attributable to the stretching modes of a specific ring, and the same results are obtained using the TB method. It is interesting to note that the signals related to the phenyl ring maintain the same position in the spectra of the complexes, not being involved in coordination bonds. On the other hand, the stretching and breathing modes of the pendant pyridine shift to higher energies upon coordination (about 20 cm^−1^ for breathing modes at 1000 cm^−1^, due to the formation of a chelating ring) [69].

In the IR spectra of [Zn(**L**)Cl_2_], [Zn(**L**)(NO_3_)_2_] and [Zn(**L**)_3_][Zn(SCN)_4_], other intense signals are observable in the 800–700 cm^−1^ spectral range. These can be assigned to breathing modes, especially of the imidazopyridine unit, which shift at higher wavenumbers in the complexes (Figure S8, Supplementary Materials). Ion exchange is confirmed by the presence of peculiar signals in the IR spectra. In particular, for [Zn(**L**)(NO_3_)_2_], it is possible to recognize the intense signals characteristic of chelating nitrate groups [70]: at 1495 cm^−1^, the ν_1_ mode of the N=O bond; at 1281 and 1265 cm^−1^, the ν_5_ asymmetric modes of the chelating N–O bond. The signal attributable to the related ν_2_ symmetric mode at 1028 cm^−1^ is Raman active. These signals are not present in the spectrum of [Zn(**L**)_3_][Zn(SCN)_4_], in which the most intense signals are attributable to the stretching mode of the C≡N bond, at a higher energy with respect to KSCN. Four signals were calculated by the semiempirical TB method and were observed in the IR spectrum (2009, 2088, 2077, 2068 cm^−1^), while in the Raman spectrum, two weak signals were detected (in Figure S9 in the Supplementary Materials). This could be related to the crystal packing: the unit cell of [Zn(**L**)_3_][Zn(SCN)_4_] contains two molecules of an anionic complex, related by an inversion centre (the only symmetry element of the lattice); thus, the mutual exclusion rules could be valid.

In the far-IR, below 300 cm^−1^, it is possible to observe the vibrational modes involving the Zn atom. In particular, the signals of Zn–N are expected at about 200 cm^−1^. Indeed, two weak signals can be observed for mono-chelate complexes (Figure S10, Supplementary Materials), due to the tetrahedral geometry. In the spectrum of the tris-chelate complex, these signals became broader and more intense. It was also possible to observe the signals of the ancillary anionic ligands. Two intense bands at 331 and 318 cm^−1^ were present in the spectrum of [Zn(**L**)Cl)_2_], attributable to Zn–Cl stretching [63], while one broad band with maxima at 280 and 265 cm^−1^ attributable to Zn–O modes [71] was observable when chloride ions are exchanged with nitrate. For [Zn(**L**)_3_][Zn(SCN)_4_], a strong and triply degenerate signal due to the stretching mode of Zn–NCS with T_d_ symmetry was observed, as expected [72].

As discussed above, no peculiar difference can be observed in the vibrational spectra of mono-, bis-, and tris-chelate complexes, so the identification of the product obtained by the reaction between Zn(NO_3_)_2_ and **L** was performed using the intense signals of nitrate in the IR spectrum (Figure S11, Supplementary Materials). The assignment was difficult due to superimposition with **L** signals: for free nitrate groups, intense asymmetric ν_1_ modes are reported below 1400 cm^−1^ [70]. In the spectrum of [Zn(**L**)_2_(NO_3_)]NO_3_, a broad and complex band was observed in the 1500–1250 cm^−1^ region, due to the presence of chelating and non-coordinated nitrate groups.

### 2.5. Mass Spectrometry

The complexes bearing one **L** ligand are neutral, and hence the corresponding mass spectra do not feature the molecular ions. In addition, these complexes do not display free basic sites where cations usually present in the ESI environment (such as Na^+^, K^+^, or NH_4_^+^) may bind and impart a positive charge. However, the signals of the metal–ligand complexes are present in the ESI spectra of each product, while the other peaks are attributed to reactions taking place in the gas-phase environment of the ESI source.

**Figure 8:**
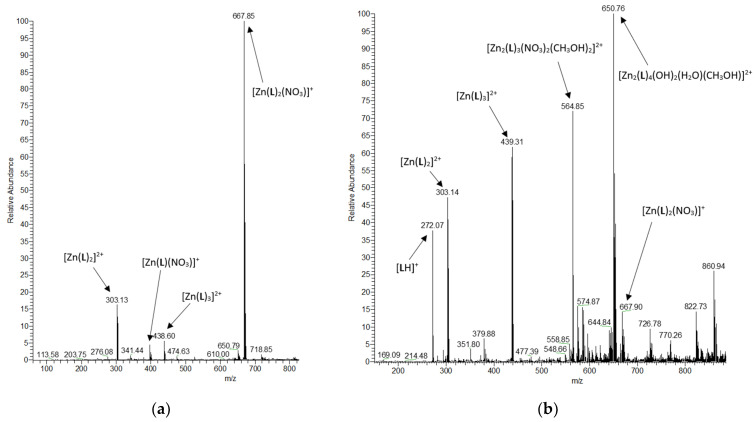
Positive ionization mass spectra of [Zn(**L**)(NO_3_)_2_] (**a**) and [Zn(**L**)_2_(NO_3_)](NO_3_) (**b**).

In Figure 8, the positive ionization mass spectra of the complexes [Zn(**L**)(NO_3_)_2_] and [Zn(**L**)_2_(NO_3_)](NO_3_) are reported. In the case of [Zn(**L**)(NO_3_)_2_] (Figure 8a), the loss of a nitrate ligand yields the [Zn(**L**)(NO_3_)]^+^ cation, whose signal is detectable at *m*/*z* = 397. The [Zn(**L**)(NO_3_)]^+^ fragment may undergo gas-phase ligand addition, binding to a second **L** molecule and thus yielding [Zn(**L**)_2_(NO_3_)]^+^ (*m*/*z* = 668). This adduct represents the base peak in the spectrum; MS/MS experiments performed on the analogous [Zn(**L**)_2_(Cl)]^+^ ion detected in the [Zn(**L**)(Cl)_2_] system show that it easily loses an **L** ligand, thus suggesting that it is a typical gas-phase product and not an ion already existing in solution (see Supplementary Materials, Figure S3). Hence, the other signals in the spectrum may be attributed to nitrate/**L** ligand exchange starting from [Zn(**L**)(NO_3_)]^+^ and yielding [Zn(**L**)_2_]^2+^ (*m*/*z* = 303, Figure 8a); this latter ion may in turn react to add a third **L** molecule, thus forming [Zn(**L**)_3_]^2+^ (*m*/*z* = 439, Figure 8a). The attribution of the doubly charged [Zn(**L**)_2_]^2+^ and [Zn(**L**)_3_]^2+^ cations was confirmed by high-resolution experiments. On the other hand, the ionic complex [Zn(**L**)_2_(NO_3_)]^+^ is clearly detectable at *m*/*z* = 668 (Figure 8b). Moreover, processes taking place in the gas phase may lead to free ligand protonation (**L**H^+^, *m*/*z* = 272) and to the [Zn(**L**)_2_]^2+^ (*m*/*z* = 303) and [Zn(**L**)_3_]^2+^ (*m*/*z* = 439) ions. The [Zn(**L**)_2_]^2+^ ion likely arises from **L** loss from the parent ion, and subsequent nitrate/**L** ligand exchange. The latter may be formed by **L** addition to the first ion or by nitrate/**L** ligand exchange from the parent [Zn(**L**)_2_(NO_3_)]^+^ ion. Condensation reactions also involving solvent molecules, which typically occur in the gas phase under ESI conditions, lead to the bulky ions [Zn_2_(**L**)_3_(CH_3_OH)_2_(NO_3_)_2_]^2+^ (*m*/*z* = 564) and [Zn_2_(**L**)_4_(OH)_2_(H_2_O)(CH_3_OH)]^2+^ (*m*/*z* = 650).

The mass spectra of the tris-chelate thiocyanate complex [Zn(**L**)_3_][Zn(SCN)_4_] (see Supplementary Materials, Figure S6) clearly feature the tris-chelate cationic form [Zn(**L**)_3_]^2+^ (*m*/*z* = 439) and the ion fragment [Zn(**L**)_2_]^2+^ (*m*/*z* = 303). Conceivable ligand exchange occurring in the gas phase between the cationic and anionic counterparts of the sample likely yields the mono-chelate ion [Zn(**L**)(SCN)]^+^ (*m*/*z* = 393). Moreover, the protonated free ligand is observed (**L**H^+^, *m*/*z* = 272).

The mass spectrum of the complex [Zn(**L**)Cl_2_] has been previously reported [63]. The molecular ion is absent; the main peaks are attributable to [Zn(**L**)_2_]^2+^ (*m*/*z* = 303), [Zn(**L**)_3_]^2+^ (*m*/*z* = 439), and [Zn(**L**)_2_Cl]^+^ (*m*/*z* = 641), which originate in ligand addition processes from [Zn(**L**)Cl]^+^ (*m*/*z* = 370) after the loss of the chloride (see Supplementary Materials, Figures S2 and S3).

The experimental mass distributions for all the observed ions fit well with the calculated isotopic mass distribution, thus representing additional proof for the assignment.

## 3. Materials and Methods

### 3.1. Experimental Techniques

All solvents and raw materials were used as received from commercial suppliers (Sigma-Aldrich, Saint Louis, MO, USA and Alfa Aesar, Ward Hill, MA, USA) without further purification. TLC was performed on a Fluka silica gel TLC-PET foils GF 254, particle size 25 nm, medium pore diameter 60 Å. Column chromatography was performed on a Sigma-Aldrich silica gel 60 (70–230 mesh ASTM).

Mass spectra were recorded on a Thermo-Finnigan Advantage Max Ion Trap Spectrometer equipped with an electrospray ion source (ESI) in positive and negative ion acquiring mode.

UV-Vis absorption spectra were recorded on a Cary60 spectrometer. Photoemission spectra were acquired with a HORIBA Jobin Yvon IBH Fluorolog-TCSPC spectrofluorometer. The spectral response was corrected for the spectral sensitivity of the photomultiplier.

FT-Raman spectra were obtained with a Bruker Vertex 70 spectrometer, equipped with the RAMII accessory, by excitation with a 1064 nm laser, with a resolution of 4 cm^−1^. FTIR spectra acquired in attenuated total reflectance (ATR) and far-infrared spectroscopy (FIR) spectra were recorded in the 4000–0 cm^−1^ range using a Bruker Vertex 70 spectrophotometer, equipped with a Harrick MVP2 ATR cell and DTGS detectors (either with Si or KBr beamsplitters). The adopted resolution was equal to 4 cm^−1^ in all cases.

The single-crystal data were collected with a Gemini R Ultra diffractometer with graphite-monochromated Mo-Kα radiation (λ = 0.71073 Å) or Cu-Kα radiation (λ = 1.5406 Å, used in the case of smaller crystals) by the ω-scan method. The cell parameters were retrieved with the CrysAlis Pro software, and the same program was used to perform data reduction with correction for the Lorentz effect and polarizing effect. Scaling and absorption corrections were applied through the CrysAlis Pro multiscan technique. All structures were solved with direct methods by using SHELXS-14 [73] and refined with the full-matrix least-squares techniques on F^2^ with SHELXL-14. All non-hydrogen atoms were refined anisotropically. Hydrogen atoms were placed in geometrical positions and refined using the riding model. Visualization of crystal structures was performed using Mercury. The crystallographic data for **L**, [Zn(**L**)(NO_3_)_2_], [Zn(**L**)_2_(NO_3_)](NO_3_), [Zn(**L**)_3_][Zn(SCN)_4_], [Zn(**L**)_2_(SCN)_2_]·DMF and [Zn(**L**)_2_(H_2_O)_2_](NO_3_)_2_ are deposited within the Cambridge Crystallographic Data Centre as supplementary publications under the CCDC numbers 2254231 and 2254151–2254155. This information can be obtained free of charge from the Cambridge Crystallographic Data Centre via www.ccdc.cam.ac.uk/data_request/cifcode CCDC (accessed on 10 April 2023). Details of structural refinement and crystallographic tables are reported in the Supplementary Materials.

Correspondence between the bulk and SC-XRD structures, as well as sample purity, was checked by comparison of the experimental and calculated powder XRD (PXRD) patterns (see Figures S18–S20 in the Supplementary Materials). Powder data were collected using an X’Pert powder diffractometer operating in a Bragg–Brentano geometry, equipped with a graphite crystal monochromator and using Cu–Kα radiation.

### 3.2. Syntheses

The samples of 3-phenyl-1-(pyridin-2-yl)imidazo[1,5-*a*]pyridine (**L**) were prepared as previously reported: [46,48,49,74] di(2-pyridyl)ketone (800 mg, 4.37 mmol, 1 eq), benzaldehyde (6.55 mmol, 1.5 eq), and ammonium acetate (1704 mg, 21.85 mmol, 5 eq) in glacial acetic acid (25 mL). Reaction time: 5 h at 118 °C. The acetic acid was removed by evaporation under reduced pressure. The obtained solid was dissolved in a saturated aqueous solution of Na_2_CO_3_ and the mixture extracted with CH_2_Cl_2_. The organic layer was separated and dried and the solvent evaporated under vacuum. The obtained crude product was purified via column chromatography on a silica gel (CH_2_Cl_2_-CH_3_OH 98:2) and the product isolated as a yellowish solid. Crystals suitable for SC-XRD were obtained from slow evaporation of the CH_2_Cl_2_ solution.

#### General Procedure for Zn(II) Complexes

**Scheme 2:**
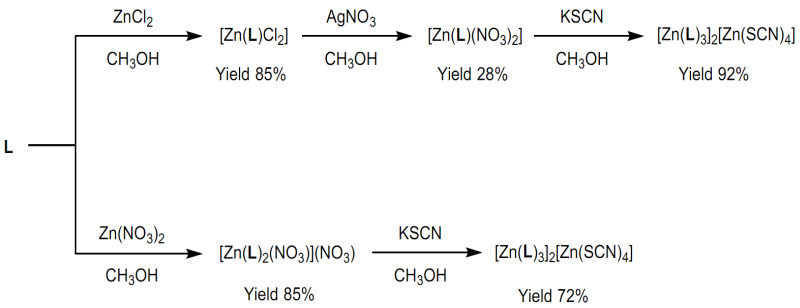
Designed synthetic strategies to obtain the mono-, bis-, and tris-chelated Zn(II) complexes by ion exchange.

[Zn(**L**)Cl_2_] was synthesized, as previously reported, [63] by reaction of a methanolic suspension (15 mL) of ZnCl_2_ (55 mg, 0.400 mmol, 1 eq) and **L** (108.5 mg, 0.400 mmol, 1 eq), at room temperature for 2.5 h. The suspension was then filtered, washed with diethyl ether, and dried under vacuum, yielding a yellow powder (yield: 138 mg, 85%, (see Scheme 2)).

[Zn(**L**)(NO_3_)_2_] was obtained by ionic exchange using AgNO_3_ (136 mg, 0.800 mmol, 2 eq), starting from [Zn(**L**)Cl_2_] (163 mg, 0.400 mmol, 1 eq) in methanol (15 mL); the reaction mixture was stirred for 15 min at room temperature. Upon reaction completion, the solution was separated from AgCl precipitate by centrifugation and then dried; the resulting yellow solid was washed three times with ice-cold water (yield: 52 mg, 28%). Crystals suitable for SC-XRD were obtained from slow evaporation of the methanol solution.

[Zn(**L**)_3_][Zn(SCN)_4_] was obtained employing [Zn(**L**)(NO_3_)_2_] as a substrate for further substitution: to a methanolic solution (10 mL) of [Zn(**L**)(NO_3_)_2_] (100 mg, 0.217 mmol, 1 eq), an aqueous solution (5 mL) of KSCN (42 mg, 0.434 mmol, 2 eq) was carefully added to form a precipitate; the suspension was stirred for 30 min at room temperature, filtered, and washed with ice-cold water (yield: 78 mg, 92%). Crystals suitable for SC-XRD were obtained from slow evaporation of the ethanolic solution.

[Zn(**L**)_2_(NO_3_)](NO_3_) was obtained by direct reaction between **L** and Zn(NO_3_)_2_, producing the bis-chelated complex independently from the stoichiometry of the reagents used. The optimal yield was obtained with a 1:2 stoichiometry: 55 mg (0.185 mmol, 1 eq) of Zn(NO_3_)_2_ and 100 mg (0.370 mmol, 2 eq) of ligand, by stirring a methanol solution (15 mL) at room temperature for 24 h. The product was recrystallized in methanol (yield: 115 mg, 85%). Crystals suitable for SC-XRD were obtained by slow evaporation of the ethanolic solution.

[Zn(**L**)_3_][Zn(SCN)_4_] was synthesized by reaction of [Zn(**L**)_2_(NO_3_)](NO_3_) (135 mg, 0.185 mmol, 1 eq, 15 mL methanol solution) with KSCN (36 mg, 0.370 mmol, 2 eq, 15 mL water solution); a precipitate immediately formed and the mixture was stirred for 1 h at room temperature. The product was filtered and washed three times with ice-cold water (yield: 150 mg, 73%).

Single crystals of [Zn(**L**)_2_(SCN)_2_]·DMF and [Zn(**L**)_2_(H_2_O)_2_](NO_3_)_2_ were obtained respectively by slow evaporation of a DMF solution of [Zn(**L**)_3_][Zn(SCN)_4_] and of a methanol solution of [Zn(**L**)_2_(NO_3_)](NO_3_).

## 4. Conclusions

A series of new fluorescent mono-, bis-, and tris-chelated Zn(II) complexes have been synthesized and characterized structurally and electronically. The complexes were obtained employing a luminescent imidazo[1,5-*a*]pyridine chelating ligand, two different Zn(II) salts, and successive anionic exchange.

The structures of the achieved compounds have been determined by single-crystal X-ray diffraction, and the complexes have been completely characterized by optical and vibrational spectroscopies and electrospray ionization mass spectrometry. For the first time, a systematic comparison of mono-, bis-, and tris-chelated imidazopyridine complexes has been studied, showing intriguing differences in the chemical structures and optical behaviours suitable for technological applications.

The optical properties of the obtained products demonstrate little modification along the series and no tunability after ion exchange or when comparing mono-, bis-, and tris-chelated forms in solution. As previously reported, an intense increase in the quantum yield is appreciable after the complexation reaction of an imidazo[1,5-*a*]pyridine-based ligand with Zn(II), due to an important modification of the ligand conformation. Furthermore, an intense modification of the quantum yield along the series in the solid-state has been observed when comparing mono-, bis-, and tris-chelated adducts. The studied products show a decrease in the quantum yield along the series (from about 6% for the mono-chelated complexes to 2% for the bis-chelated one, and an intense decrease for the tris-chelated form (0.5%)). All the obtained products show an appreciable large Stokes shift as previously reported for similar imidazo[1,5-*a*]pyridines complexes due to the charge-transfer character centred on the employed fluorescent ligand.

In general, the obtained Zn(II) 1,3-substituted-imidazo[1,5-*a*]pyridines complexes show absorption maxima below 415 nm, with a suitable transparency in the visible range, and a powerful emission in the blue region (410–450 nm in solution and 450–550 in the solid state).

On the basis of these promising properties, further studies are in progress to test these products as low-cost emitting materials for useful technological applications such as down-shifting or down-conversion.

## Figures and Tables

**Table 1:** Absorption and emission data for **L** and for the mono-, bis-, and tris-chelate Zn(II) complexes in dichloromethane solutions (sh = shoulder).

Compound	Absorption (nm)	Excitation (nm)	Emission (nm)	Stokes Shift (nm)	Quantum Yield (%)
**L**	377 sh	377	463	79	19
	324				
[Zn(**L**)Cl_2_]	395 sh	376	462 sh	64	32
	376		440		
	362		416 sh		
[Zn(**L**)(NO_3_)_2_]	394 sh	375	462 sh 439 418 sh	64	36
	375				
	363				
	318				
	294 sh				
[Zn(**L**)_2_(NO_3_)](NO_3_)	398 sh	373	460 sh 435 415 sh	62	35
	373				
	361				
	322				
[Zn(**L**)_3_][Zn(SCN)_4_]	395 sh	374	460 sh 438	64	25
	374				
	359				
	323				
	296 sh				

**Table 2:** Absorption and emission data for **L** and for the mono-, bis-, and tris-chelate Zn(II) complexes (solid state).

Compound	Excitation (nm)	Emission (nm)	Stokes Shift (nm)	Quantum Yield (%)
**L**	425	520 sh	83	5
		491		
		460		
[Zn(**L**)Cl_2_]	418	498	142	6
[Zn(**L**)(NO_3_)_2_]	444	493	125	7
[Zn(**L**)_2_(NO_3_)](NO_3_)	425	495	105	2
[Zn(**L**)_3_][Zn(SCN)_4_]	423	475	91	<1

## Data Availability

The data presented in this study are available within the article and the Supplementary Materials.

## Supplementary Materials

The following supporting information can be downloaded at: https://www.mdpi.com/article/10.3390/molecules28093703/s1, Synthesis **L**, Figure S1: ^1^H NMR spectrum of **L** in DMSO-d6; Figure S2: Positive ionisation ESI-MS full mass spectrum of [Zn(**L**)Cl_2_]; Figure S3: Positive ionisation ESI-MS/MS mass spectrum of [Zn(**L**)_2_Cl]^+^; Figure S4: Positive ionisation ESI-MS/MS mass spectrum of [Zn(**L**)_2_(NO_3_)]^+^; Figure S5: Positive ionisation ESI-MS/MS mass spectrum of [Zn(**L**)_3_]^2+^; Figure S6: Positive ionisation ESI-MS mass spectrum of [Zn(**L**)_3_][Zn(SCN)_4_; Table S1. Raman and IR signals of **L** and Zn(II) mono- and tris-chelated complexes; Figure S7: Molecular structure and numbering of **L** for vibrational assignment; Figure S8: FTIR-ATR spectra of **L** and corresponding mono-chelated and tri-chelated complexes; Figure S9: FTIR-ATR and Raman spectra of [Zn(**L**)_3_][Zn(SCN)_4_]; Figure S10: FTIR-ATR spectra in the Far-IR region of **L** and corresponding mono-chelated and tri-chelated complexes; Figure S11: FTIR-ATR spectra of **L** and corresponding mono-chelated and bis-chelated complexes obtained using nitrate as ancillary ligand; Figure S12: Asymmetric unit of **L**; Table S2: Crystal data and structure refinement for **L**; Table S3: Bond lengths for **L**; Table S4: Bond angles for **L**; Figure S13: Asymmetric unit of [Zn(**L**)(NO_3_)_2_]; Table S5: Crystal data and structure refinement for [Zn(**L**)(NO_3_)_2_]; Table S6: Bond lengths for [Zn(**L**)(NO_3_)_2_]; Table S7: Bond angles for [Zn(**L**)(NO_3_)_2_]; Figure S14: Asymmetric unit of [Zn(**L**)_2_(NO_3_)](NO_3_)·H_2_O; Table S8: Crystal data and structure refinement for [Zn(**L**)_2_(NO_3_)](NO_3_)·H_2_O; Table S9: Bond lengths for [Zn(**L**)_2_(NO_3_)](NO_3_)·H_2_O; Table S10: Bond angles for [Zn(**L**)_2_(NO_3_)](NO_3_)·H_2_O; Figure S15: Asymmetric unit of [Zn(**L**)_2_(H_2_O)_2_](NO_3_)_2_; Table S11: Crystal data and structure refinement for [Zn(**L**)_2_(H_2_O)_2_](NO_3_)_2_; Table S12: Bond lengths for [Zn(**L**)_2_(H_2_O)_2_](NO_3_)_2_; Table S13: Bond angles for [Zn(**L**)_2_(H_2_O)_2_](NO_3_)_2_; Figure S16: Asymmetric unit of [Zn(**L**)_3_][Zn(SCN)_4_]; Table S14: Crystal data and structure refinement for [Zn(**L**)_3_][Zn(SCN)_4_]; Table S15: Bond lengths for [Zn(**L**)_3_][Zn(SCN)_4_]; Table S16: Bond angles for [Zn(**L**)_3_][Zn(SCN)_4_]; Figure S17: Asymmetric unit of [Zn(**L**)_2_(SCN)_2_]·DMF; Table S17: Crystal data and structure refinement for [Zn(**L**)_2_(SCN)_2_]·DMF; Table S18: Bond lengths for [Zn(**L**)_2_(SCN)_2_]·DMF; Table S19: Bond angles for [Zn(**L**)_2_(SCN)_2_]·DMF; Figure S18: Experimental and calculated PXRD pattern of [Zn(**L**)(NO_3_)_2_]; Figure S19: Experimental pattern of direct reaction between Zn(NO_3_)_2_ and **L**, and calculated PXRD patterns of [Zn(**L**)_2_(NO_3_)](NO_3_)∙H_2_O and [Zn(**L**)(NO_3_)_2_]; Figure S20: Experimental and calculated PXRD pattern of [Zn(**L**)_3_][Zn(SCN)_4_].
