# Supplementary material for: Mono-, Bis-, and Tris-Chelate Zn(II) Complexes with Imidazo[1,5-a]pyridine: Luminescence and Structural Dependence
Source: Molecules. 2023 Apr 25;28(9):3703. doi: 10.3390/molecules28093703 (PMC10179938; doi:10.3390/molecules28093703)
Supplement: Supplementary file 1 [file molecules-28-03703-s001.zip › molecules-2367714-supplementary.pdf]

Mono-, bis- and tris-chelate Zn(II) complexes with imidazo[1,5-*a*]pyridine: luminescence and structural dependence

Valerio Cerrato, Giorgio Volpi,* Emanuele Priola, Alessia Giordana, Claudio Garino,* Roberto Rabezzana, Eliano Diana.

Department of Chemistry, University of Turin, Via Pietro Giuria 7, 10125, Torino, Italy

Keywords: imidazo[1,5-*a*]pyridine, luminescence, fluorescence, zinc complex, quantum yield, Stokes shift.

Synthesis of 3-phenyl-1-(pyridin-2-yl)imidazo[1,5-*a*]pyridine (L)

2,2′-dipyridyl ketone (800 mg, 4.37 mmol, 1 eq), benzaldehyde (6.55 mmol, 1.5 eq) and ammonium acetate (1704 mg, 21.85 mmol, 5 eq) are put in a 50 ml round-bottom flask (the large excess of aldehyde is needed to minimise the amount of unreacted ketone). Then 15 ml of glacial acetic acid is added as solvent. The reaction proceeds under reflux (T = 118 °C) for 12 hours. The reaction progress is monitored via thin-layer chromatography. Yield: 69%.

Mass (ESI +): *m*/*z* = 272.21, corresponding to the protonated molecule.

^{1}H NMR spectroscopy (600 MHz, DMSO-d6): δ 8.59 (1H, d, *J* = 9.0 Hz), 8.48 (1H, d, *J* = 4.5 Hz), 8.10 (1H, d, *J* = 7.5 Hz), 7.85 (2H, d, *J* = 7.5 Hz), 7.79 (1H, t, *J* = 7.5 Hz), 7.56 (3H, t, *J* = 7.5 Hz), 7.49 (1H, t, *J* = 7.5 Hz), 7.16 (1H, dd, *J* = 6.5 Hz), 7.03 (1H, dd, *J* = 9 Hz), 6.82 (1H, t, *J* = 6.5 Hz).

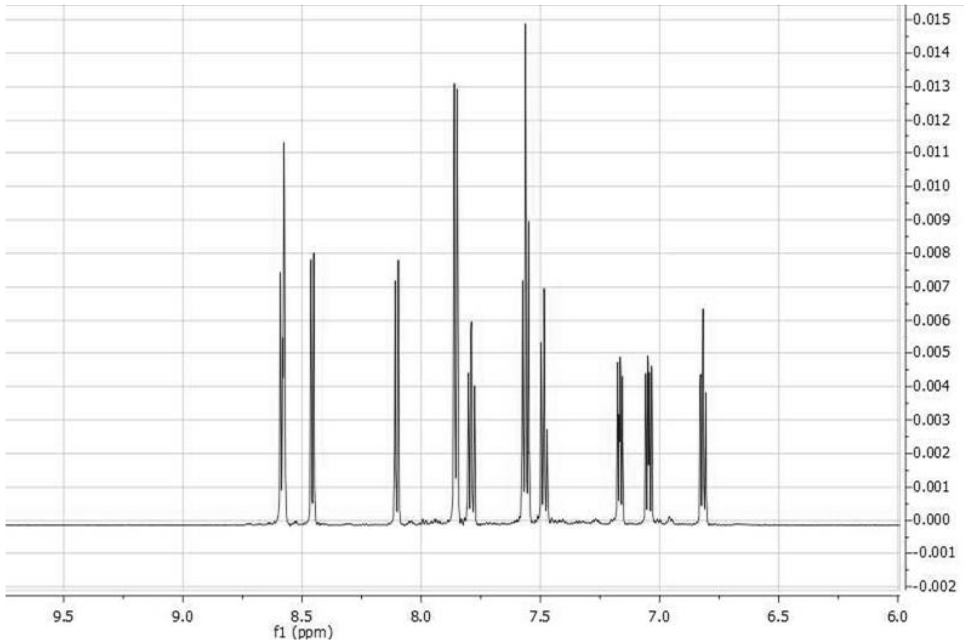

Figure S1. ^{1}H NMR spectrum of 3-phenyl-1-(pyridin-2-yl)imidazo[1,5-*a*]pyridine (**L)** in DMSO-d6.

Mass Spectra from methanolic solutions:

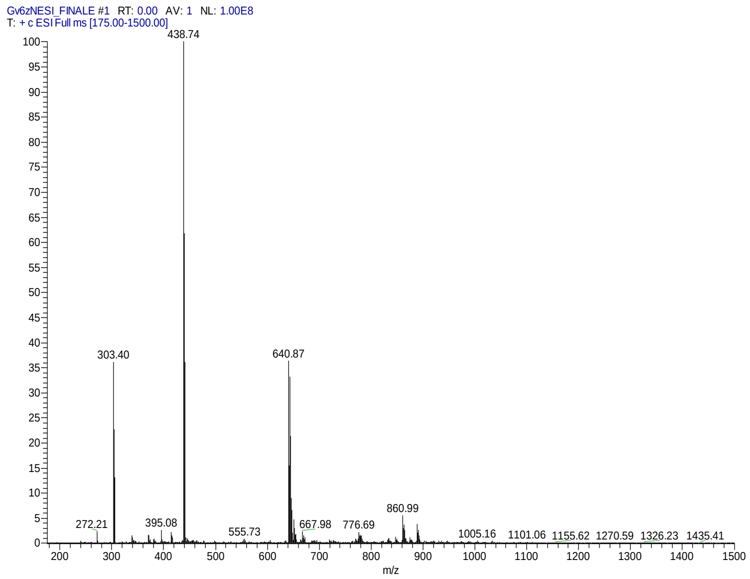

Figure S2. Positive ionisation ESI-MS full mass spectrum of complex [Zn(**L**)Cl_2].

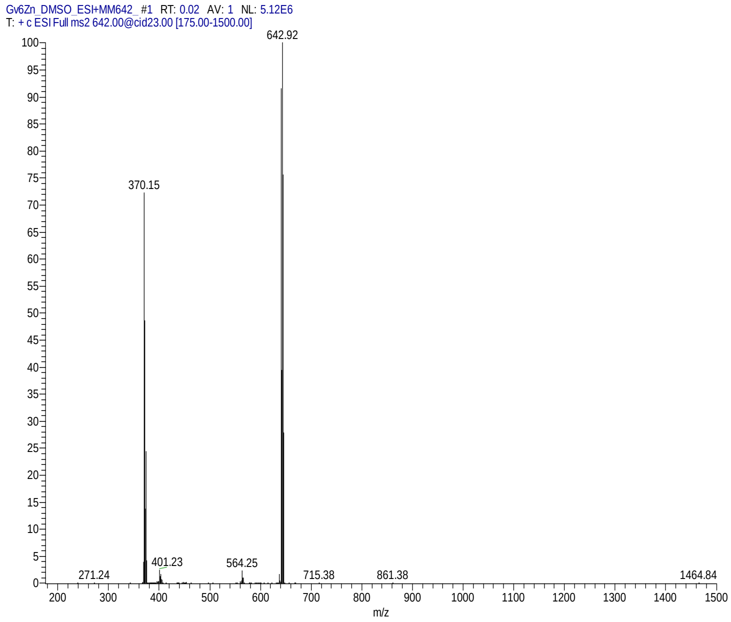

Figure S3. Positive ionisation ESI-MS/MS mass spectrum of the ion complex [Zn(**L**)$_2$Cl]$^+$ (m/z = 641), formed by ionization of [Zn(**L**)Cl_2].

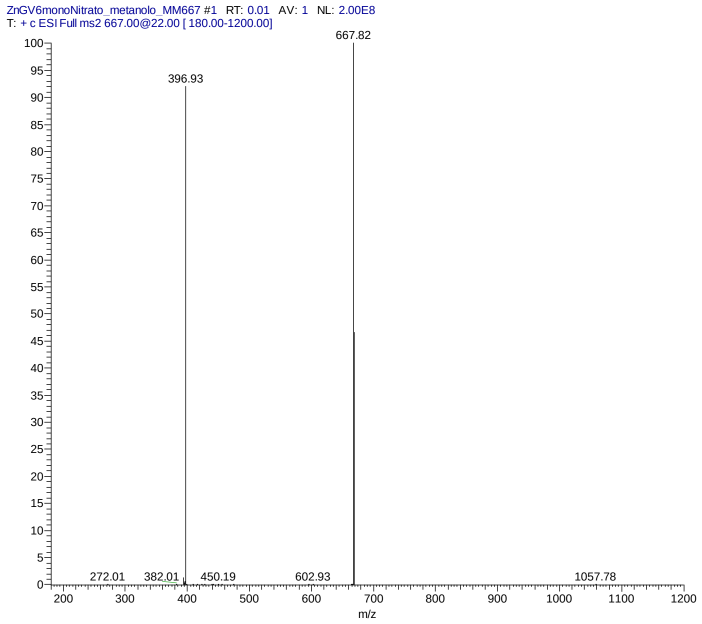

Figure S4. Positive ionisation ESI-MS/MS mass spectrum of complex $[Zn(\mathbf{L})_2(NO_3)]^+$ (m/z = 668), formed by ionisation of $[Zn(\mathbf{L})(NO_3)_2]$.

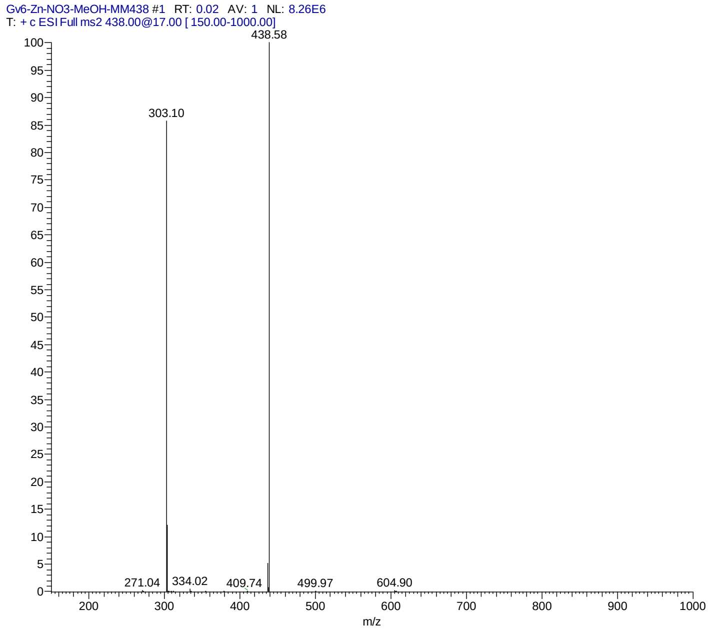

Figure S5. Positive ionisation ESI-MS/MS mass spectrum of complex $[Zn(\mathbf{L})_3]^{2+}$ (m/z = 439), formed by ionisation of $[Zn(\mathbf{L})(NO_3)_2]$.

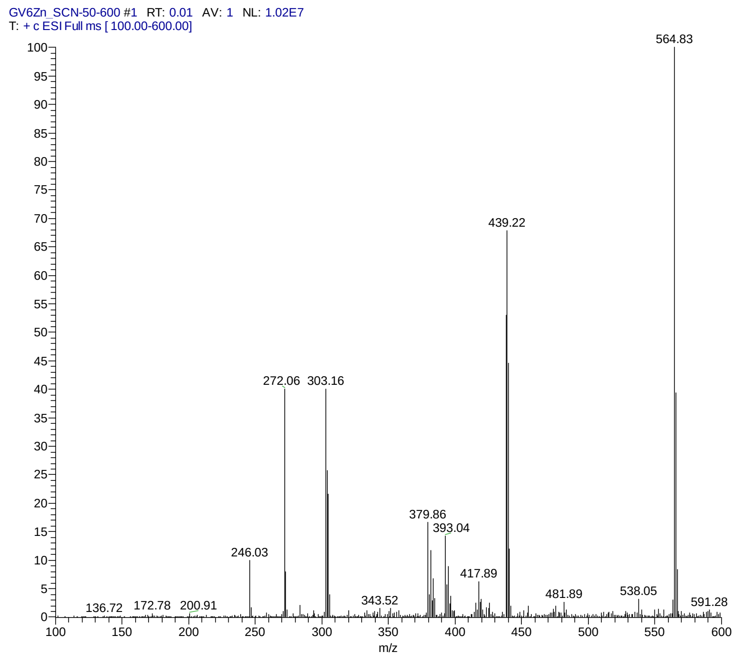

Figure S6. Positive ionisation ESI-MS mass spectrum of complex $[Zn(\mathbf{L})_3][Zn(SCN)_4]$.

Table S1. Raman and IR signals of **L** and Zn(II) mono- and tris-chelated complexes.

**L**		[Zn(**L**)(Cl)$_2$]		[Zn(**L**)(NO_3)$_2$]		[Zn(**L**)$_3$][Zn(SCN)$_4$]		
Raman	IR	Raman	IR	Raman	IR	Raman	IR	
						2110 w<br>2077 w	2109 w<br>2085 sh<br>2077 sh<br>2068 vvs	ν C≡N
1631 m	1634 w	1638 w	1649 w	1643 w	1642 w	1638 w	1638 w	ν ring B
1603 s	1602 w	1604 vs	1609 s	1607 s	1610 m			ν ring D
1588 s	1584 s		1602 sh		1600 m	1607 s	1607 s	ν ring C
		1589 sh	1589 sh				1588 sh	ν ring D[a]
1563 w	1562 m	1562 w	1563 w	1567 w	1566 vw	1568 w	1565 w	ν C=N + ν C=C
1533 vs	1532 m	1547 s	1549 m	1552 vs	1551 m	1551 w	1548 m	ν ring A+B
1523 s	1524 m	1533 s	1535 m	1537 vs	1536 w	1534 vs	1532 m	ν C1–C ring D
1507 s	1508 s	1513 vs	1515 s	1518 s	1518 sh<br>1510 s	1515 m	1512 s	ν ring A+B + ν C2–C ring C
					1495 s			ν N=O
1460 m	1465 sh	1478 w	1479 s	1479 w	1476 vs	1482 w	1479 s	ν C=C + δ CCH
1443 m<br>1428 m	1443 m<br>1427 m	1447 m<br>1436 m	1448 m<br>1438 s	1449 m<br>1436 m	1448 s<br>1435 s	1442 m	1446 m<br>1436 m	δ CCH + δ CCC
1404 w	1403 m	1408 m		1411 m		1410 w	1409 vw	δ C=N
1354 s	1354 m	1369 m	1370 m	1369 m	1367 m	1363 m	1358 w	ν ring A + δ CCH
1333 w	1334 w	1342 w	1342 m	1342 sh	1342 m	1335 m		δ CCH
1317 w	1317 m	1329 m<br>1317 w	1330 sh<br>1317 w	1332 s<br>1316 w	1316 w		1331 m	Kekulé mode ring D, B, A
1277 vw	1277 m	1285 w	1287 w	1287 w	1294 m	1281 w	1281 w	Kekulé mode ring C
					1218 s<br>1265 s			ν_a (NO_2)
1245 m	1247 m	1256 m	1255 w	1255 w	1250 sh	1251 w	1251 w	ν ring A + δ CCH
1008 w<br>998 m<br>980 s	1008 m<br>997 w<br>980 w<br>950 m	1023 m<br>1012 m<br>994 s	1032 w<br>1025 vw<br>1014 m<br>997 w	1027 s*<br>1015 m<br>997 s	1035 vw<br>1012 m<br>995 w	1021 w<br>997 m	1031 w<br>1008 m<br>993 m	Breathing mode<br>* ν_s (NO_2)
790 vvw	786 m		795 m<br>783 s<br>770 m		794 w<br>780 m<br>769 w		791 sh<br>781 s<br>763 m	Breathing mode

L		$[Zn(\mathbf{L})(Cl)_2]$		$[Zn(\mathbf{L})(NO_3)_2]$		$[Zn(\mathbf{L})_3][Zn(SCN)_4]$		
Raman	IR	Raman	IR	Raman	IR	Raman	IR	
747 w<br><br>729 vvw<br>702 vvw	746 m<br>737 m<br>729 m<br>700 m		752 sh<br>747 s<br>732 w		752 w<br>737 m		748 m<br>736 m	γ CCH
685 vw	691 s		697 vs		696 s		698 s	Breathing mode A+B rings
		340 m		350 w		340 w		Ligand mode
			331 s<br>308 s					Zn–Cl
					282 m<br>265 m			Zn–O
						270 w	270 m, br	Zn–N (SCN)
		170 w	170 w	175 w	175w		172 m	Zn–N (**L**)?
s=strong, m=medium, w=weak, v=very, sh=shoulder, br=broad, ν=stretching, δ=bending, γ=out-of-plane deformation, numbering as indicate in Figure S7								

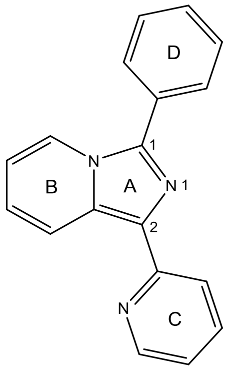

Figure S7. Molecular structure and numbering of **L** for vibrational assignment.

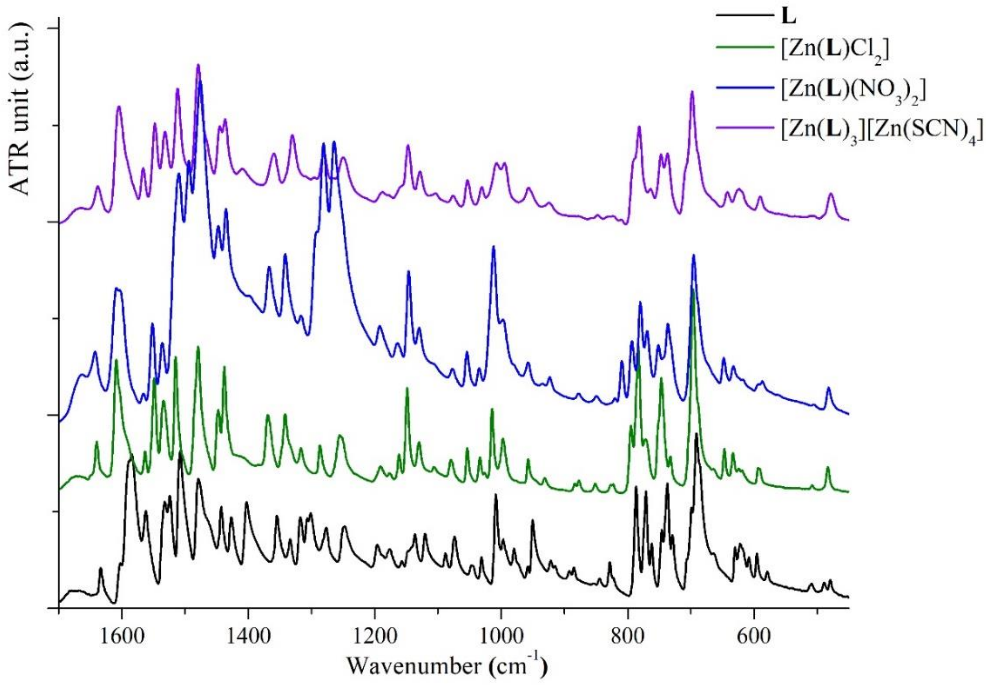

Figure S8. FTIR-ATR spectra of ligand **L** and corresponding mono-chelated and tri-chelated complexes.

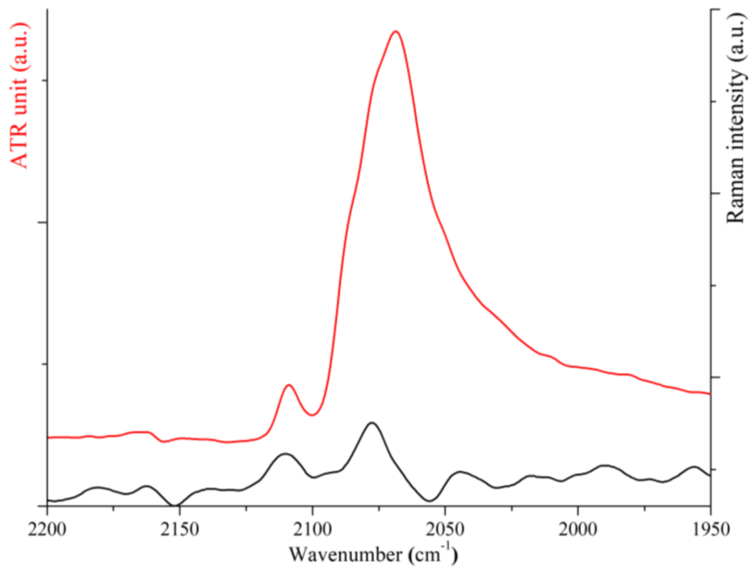

Figure S9. FTIR-ATR (red) and Raman (black) spectra of [Zn(**L**)$_3$][Zn(SCN)$_4$].

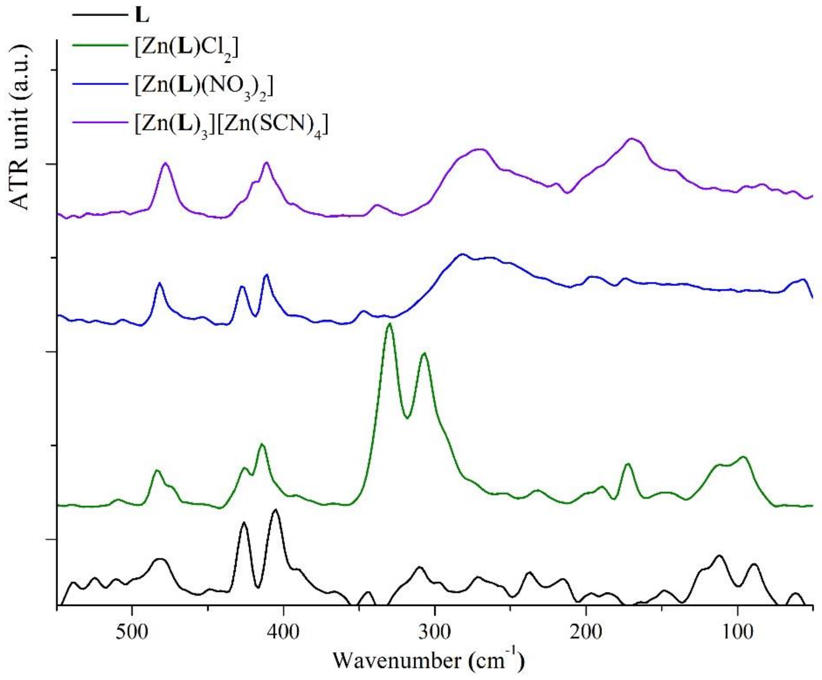

Figure S10. FTIR-ATR spectra in the Far-IR region of ligand **L** and corresponding mono-chelated and tri-chelated complexes.

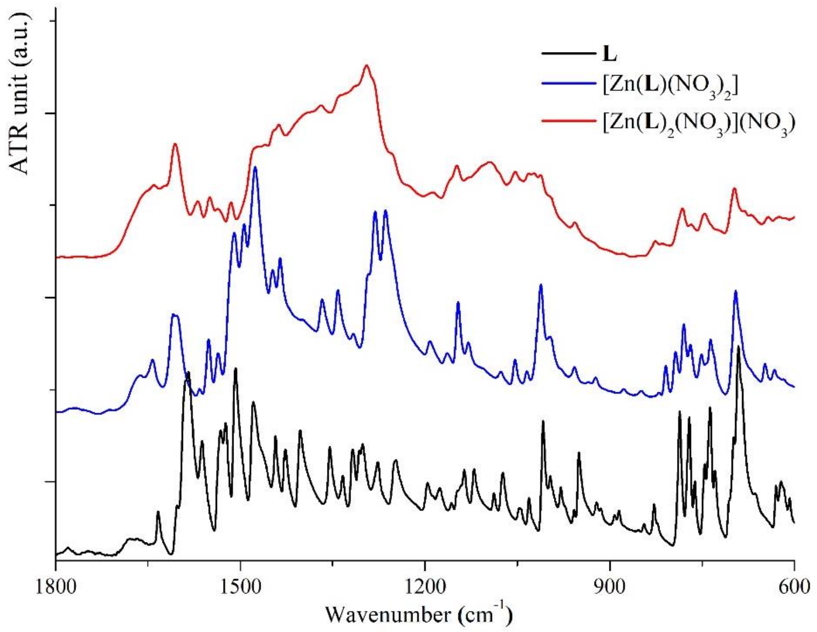

Figure S11. FTIR-ATR spectra of ligand **L** and corresponding mono-chelated and bis-chelated complexes obtained using nitrate as ancillary ligand.

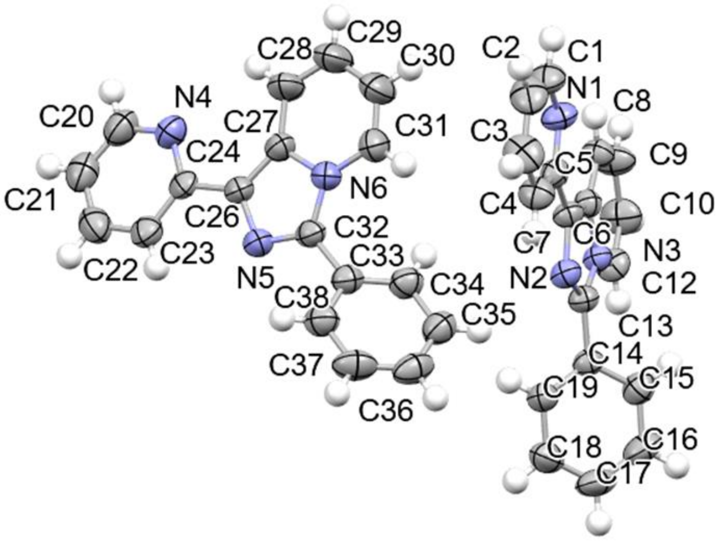

Figure S12. Asymmetric unit of **L**.

Table S2. Crystal data and structure refinement for **L**.

Empirical formula	$C_{18}H_{13}N_3$
Formula weight	271.31
Temperature/K	293.00
Crystal system	triclinic
Space group	P-1
a/Å	9.0118(16)
b/Å	10.5527(15)
c/Å	15.107(2)
α/°	95.821(12)
β/°	98.725(14)
γ/°	96.787(14)
Volume/Å^3	1399.6(4)
Z	4
ρ_{calc}g/cm^3	1.288
μ/mm^{-1}	0.078
F(000)	568.0
Crystal size/mm^3	0.11 × 0.1 × 0.08
Radiation	MoKα (λ = 0.71073)
2Θ range for data collection/°	6.35 to 59.078
Index ranges	-11 ≤ h ≤ 11, -10 ≤ k ≤ 14, -18 ≤ l ≤ 20
Reflections collected	12738
Independent reflections	6435 [R_{int} = 0.0904, R_{sigma} = 0.2429]
Data/restraints/parameters	6435/0/379
Goodness-of-fit on F^2	0.945
Final R indexes [I>=2σ (I)]	R_1 = 0.0936, wR_2 = 0.1508
Final R indexes [all data]	R_1 = 0.3034, wR_2 = 0.3884
Largest diff. peak/hole / e Å^{-3}	0.15/-0.16

Table S3. Bond lengths for **L**.

Atom	Atom	Length/Å		Atom	Atom	Length/Å
N3	C13	1.380(4)		N6	C32	1.376(4)
N3	C7	1.404(4)		N6	C31	1.396(4)
N3	C12	1.371(4)		C19	C18	1.386(5)
N2	C6	1.375(4)		C33	C34	1.385(5)
N2	C13	1.318(5)		C33	C32	1.472(5)
N5	C26	1.380(4)		C33	C38	1.375(5)
N5	C32	1.323(4)		C12	C10	1.346(5)
C6	C7	1.379(5)		C23	C22	1.386(5)
C6	C5	1.451(5)		C34	C35	1.387(5)
C27	C26	1.375(5)		C35	C36	1.377(6)
C27	N6	1.403(4)		C31	C30	1.329(5)

C27	C28	1.405(5)		C8	C9	1.344(5)
C13	C14	1.475(5)		C18	C17	1.363(6)
C7	C8	1.413(5)		C17	C16	1.366(6)
C26	C24	1.461(5)		C10	C9	1.413(5)
C5	N1	1.345(4)		C3	C4	1.375(5)
C5	C4	1.389(5)		C3	C2	1.370(5)
N1	C1	1.341(5)		C1	C2	1.375(6)
C14	C15	1.389(5)		C20	C21	1.358(5)
C14	C19	1.368(5)		C21	C22	1.372(5)
C15	C16	1.390(5)		C36	C37	1.367(6)
C24	N4	1.349(4)		C28	C29	1.345(5)
C24	C23	1.378(5)		C30	C29	1.415(5)
N4	C20	1.331(4)		C37	C38	1.389(5)

Table S4. Bond angles for **L**.

Atom	Atom	Atom	Angle/°		Atom	Atom	Atom	Angle/°
C13	N3	C7	106.4(3)		C32	N6	C31	130.9(4)
C12	N3	C13	130.9(4)		C31	N6	C27	121.6(3)
C12	N3	C7	122.5(3)		C14	C19	C18	120.4(5)
C13	N2	C6	106.7(3)		C34	C33	C32	122.0(4)
C32	N5	C26	106.3(3)		C38	C33	C34	119.5(4)
N2	C6	C7	110.2(4)		C38	C33	C32	118.5(4)
N2	C6	C5	121.2(4)		C10	C12	N3	118.4(4)
C7	C6	C5	128.5(4)		C24	C23	C22	118.7(4)
C26	C27	N6	104.9(3)		C33	C34	C35	120.2(4)
C26	C27	C28	137.8(4)		N5	C32	N6	110.9(3)
N6	C27	C28	117.3(4)		N5	C32	C33	124.2(4)
N3	C13	C14	124.3(4)		N6	C32	C33	125.0(4)
N2	C13	N3	111.3(3)		C36	C35	C34	120.1(5)
N2	C13	C14	124.3(4)		C30	C31	N6	118.6(4)
N3	C7	C8	117.8(4)		C9	C8	C7	119.1(4)
C6	C7	N3	105.5(3)		C17	C18	C19	120.3(5)
C6	C7	C8	136.8(4)		C18	C17	C16	120.1(5)
N5	C26	C24	121.2(4)		C12	C10	C9	120.8(4)
C27	C26	N5	110.6(3)		C2	C3	C4	119.3(5)
C27	C26	C24	128.1(4)		N1	C1	C2	124.1(4)
N1	C5	C6	117.7(4)		N4	C20	C21	125.4(4)
N1	C5	C4	122.1(4)		C20	C21	C22	117.8(4)
C4	C5	C6	120.2(4)		C37	C36	C35	119.6(4)
C1	N1	C5	117.0(4)		C3	C4	C5	119.2(4)
C15	C14	C13	121.6(4)		C8	C9	C10	121.3(4)

C19	C14	C13	119.1(4)		C29	C28	C27	120.9(4)
C19	C14	C15	119.3(4)		C3	C2	C1	118.2(5)
C14	C15	C16	119.7(4)		C31	C30	C29	121.8(4)
N4	C24	C26	116.5(4)		C21	C22	C23	119.2(4)
N4	C24	C23	122.5(4)		C17	C16	C15	120.2(5)
C23	C24	C26	120.9(4)		C36	C37	C38	120.8(5)
C20	N4	C24	116.4(4)		C28	C29	C30	119.7(4)
C32	N6	C27	107.2(3)		C33	C38	C37	119.9(5)

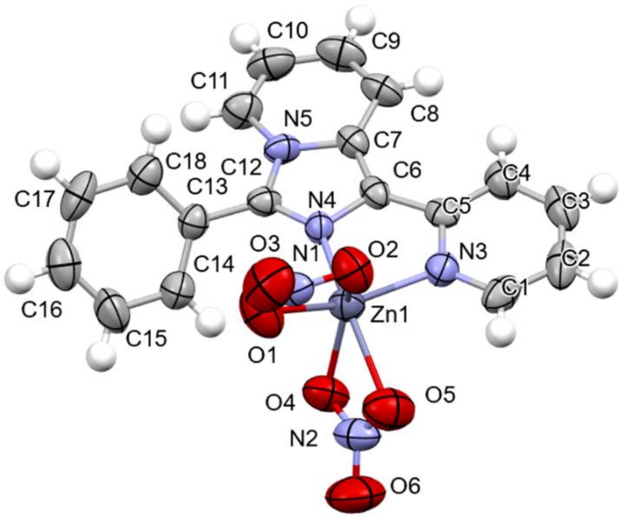

Figure S13. Asymmetric unit of [Zn(**L**)(NO_3)$_2$].

Table S5. Crystal data and structure refinement for [Zn(**L**)(NO_3)$_2$].

Empirical formula	$C_{18}H_{13}N_5O_6Zn$
Formula weight	460.73
Temperature/K	298.00
Crystal system	monoclinic
Space group	$P2_1/n$
a/Å	14.791(3)
b/Å	8.2582(16)
c/Å	15.828(4)
α/°	90
β/°	107.40(2)
γ/°	90
Volume/Å^3	1844.8(7)
Z	4
ρ_{calc}g/cm^3	1.6587
μ/mm^{-1}	1.381

F(000)	937.7
Crystal size/mm^3	0.1 × 0.09 × 0.04
Radiation	Mo Kα (λ = 0.71073)
2Θ range for data collection/°	6.62 to 52.74
Index ranges	$-17 \le h \le 18$, $-8 \le k \le 10$, $-19 \le l \le 13$
Reflections collected	7294
Independent reflections	3615 [R_{int} = 0.1106, R_{sigma} = 0.2230]
Data/restraints/parameters	3615/0/272
Goodness-of-fit on F^2	0.926
Final R indexes [I>=2σ (I)]	R_1 = 0.0729, wR_2 = 0.2289
Final R indexes [all data]	R_1 = 0.2168, wR_2 = 0.2304
Largest diff. peak/hole / e Å^{-3}	1.75/-1.31

Table S6. Bond lengths for [Zn(**L**)(NO_3)$_2$].

Atom	Atom	Length/Å		Atom	Atom	Length/Å
Zn1	N3	2.069(5)		C13	C12	1.476(8)
Zn1	O4	2.074(5)		C13	C18	1.375(9)
Zn1	N4	2.041(6)		C13	C14	1.382(9)
Zn1	O2	2.153(5)		C7	C6	1.386(9)
Zn1	O1	2.130(5)		C11	C10	1.331(9)
Zn1	O5	2.323(6)		C1	C2	1.369(9)
N5	C7	1.402(7)		N2	O6	1.200(8)
N5	C12	1.375(8)		N2	O5	1.222(8)
N5	C11	1.383(8)		N1	O2	1.232(7)
N3	C5	1.340(8)		N1	O1	1.250(7)
N3	C1	1.340(8)		N1	O3	1.218(7)
O4	N2	1.271(8)		C4	C3	1.372(10)
C8	C7	1.425(9)		C3	C2	1.365(10)
C8	C9	1.338(10)		C17	C16	1.379(10)
N4	C12	1.309(8)		C17	C18	1.396(9)
N4	C6	1.379(7)		C16	C15	1.349(10)
C5	C6	1.457(9)		C10	C9	1.399(10)
C5	C4	1.389(8)		C15	C14	1.382(9)

Table S7. Bond angles for [Zn(**L**)(NO_3)$_2$].

Atom	Atom	Atom	Angle/°		Atom	Atom	Atom	Angle/°
O4	Zn1	N3	110.1(2)		C14	C13	C18	119.8(7)
N4	Zn1	N3	80.8(2)		C8	C7	N5	117.2(7)
N4	Zn1	O4	105.7(2)		C6	C7	N5	105.0(6)
O2	Zn1	N3	94.5(2)		C6	C7	C8	137.7(7)
O2	Zn1	O4	138.0(2)		N4	C12	N5	108.9(6)
O2	Zn1	N4	111.5(2)		C13	C12	N5	123.8(7)
O1	Zn1	N3	152.9(2)		C13	C12	N4	127.3(7)
O1	Zn1	O4	95.0(2)		C5	C6	N4	118.9(6)
O1	Zn1	N4	102.7(2)		C7	C6	N4	108.3(6)
O1	Zn1	O2	58.9(2)		C7	C6	C5	132.7(6)
O5	Zn1	N3	92.8(2)		C10	C11	N5	119.7(7)
O5	Zn1	O4	57.3(2)		C2	C1	N3	122.0(8)
O5	Zn1	N4	158.6(2)		O6	N2	O4	120.3(9)
O5	Zn1	O2	89.2(2)		O5	N2	O4	116.3(8)
O5	Zn1	O1	92.4(2)		O5	N2	O6	123.4(10)
C12	N5	C7	108.3(6)		O1	N1	O2	116.1(7)
C11	N5	C7	121.7(6)		O3	N1	O2	122.2(8)
C11	N5	C12	129.8(6)		O3	N1	O1	121.6(7)

C5	N3	Zn1	114.8(5)		N1	O2	Zn1	92.1(5)
C1	N3	Zn1	125.8(5)		C3	C4	C5	117.5(8)
C1	N3	C5	119.3(6)		N1	O1	Zn1	92.6(4)
N2	O4	Zn1	98.4(5)		C2	C3	C4	121.1(7)
C9	C8	C7	118.8(7)		C18	C17	C16	119.7(8)
C12	N4	Zn1	139.1(5)		C15	C16	C17	120.3(8)
C6	N4	Zn1	111.5(5)		N2	O5	Zn1	87.9(5)
C6	N4	C12	109.4(6)		C17	C18	C13	119.5(8)
C6	C5	N3	113.7(6)		C9	C10	C11	119.9(8)
C4	C5	N3	121.7(7)		C3	C2	C1	118.4(8)
C4	C5	C6	124.6(7)		C14	C15	C16	120.7(8)
C18	C13	C12	121.1(7)		C15	C14	C13	119.8(8)
C14	C13	C12	118.9(7)		C10	C9	C8	122.6(8)

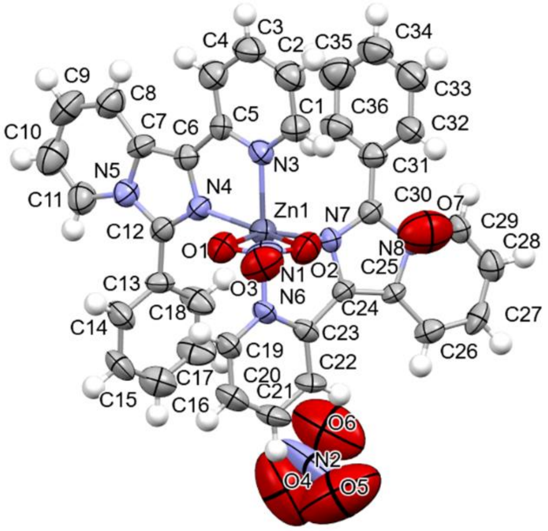

Figure S14. Asymmetric unit of $[Zn(\mathbf{L})_2(NO_3)](NO_3)\cdot H_2O$.

Table S8. Crystal data and structure refinement for $[Zn(\mathbf{L})_2(NO_3)](NO_3)\cdot H_2O$.

Empirical formula	$C_{36}H_{26}N_8O_7Zn$
Formula weight	748.02
Temperature/K	298
Crystal system	triclinic
Space group	P-1
a/Å	10.9841(14)
b/Å	11.998(2)
c/Å	14.8171(15)
α/°	85.369(11)
β/°	89.737(9)
γ/°	68.886(13)

Volume/$Å^3$	1815.0(4)
Z	2
ρ_{calc}g/cm^3	1.369
μ/mm^{-1}	1.439
F(000)	768.0
Crystal size/mm^3	0.15 × 0.12 × 0.11
Radiation	CuKα (λ = 1.54184)
2Θ range for data collection/°	7.928 to 135.944
Index ranges	-13 ≤ h ≤ 13, -14 ≤ k ≤ 14, -17 ≤ l ≤ 17
Reflections collected	6684
Independent reflections	6607 [R_{int} = 0.0858, R_{sigma} = 0.0932]
Data/restraints/parameters	6607/126/469
Goodness-of-fit on F^2	0.970
Final R indexes [I>=2σ (I)]	R_1 = 0.0446, wR_2 = 0.0816
Final R indexes [all data]	R_1 = 0.0673, wR_2 = 0.0929
Largest diff. peak/hole / e $Å^{-3}$	0.21/-0.29

Table S9. Bond lengths for [Zn(**L**)$_2$(NO_3)](NO_3)·H_2O.

Atom	Atom	Length/Å		Atom	Atom	Length/Å
Zn1	N6	2.111(6)		C35	C34	1.359(10)
Zn1	N3	2.118(6)		C31	C32	1.374(9)
Zn1	O1	2.253(6)		C18	C17	1.379(10)
Zn1	O2	2.293(6)		C18	C13	1.378(9)
Zn1	N4	2.049(6)		C34	C33	1.360(10)
Zn1	N7	2.061(6)		N5	C12	1.365(9)
C20	C21	1.371(10)		N5	C11	1.384(9)
C20	C19	1.374(9)		N5	C7	1.394(8)
N6	C19	1.331(9)		C12	C13	1.466(11)
N6	C23	1.361(9)		C11	C10	1.341(10)
N3	C5	1.327(8)		C8	C9	1.344(10)
N3	C1	1.336(8)		C8	C7	1.396(10)
O1	N1	1.273(9)		C9	C10	1.419(10)
C6	C5	1.450(10)		C16	C17	1.348(10)
C6	N4	1.378(8)		C16	C15	1.362(10)
C6	C7	1.373(10)		C13	C14	1.375(9)
N1	O3	1.232(8)		C14	C15	1.389(10)
N1	O2	1.265(8)		C28	C29	1.321(9)
C5	C4	1.395(9)		C28	C27	1.413(10)
N8	C30	1.354(8)		C21	C22	1.353(9)
N8	C29	1.362(9)		C24	C23	1.467(10)
N8	C25	1.401(8)		C24	C25	1.376(10)
C3	C4	1.397(9)		C24	N7	1.391(8)

C3	C2	1.356(9)		C22	C23	1.383(9)
C30	C31	1.475(10)		C26	C27	1.352(9)
C30	N7	1.325(8)		C26	C25	1.409(9)
C36	C35	1.396(10)		C32	C33	1.402(9)
C36	C31	1.368(10)		O5	N2	1.23(5)
N4	C12	1.325(9)		O4	N2	1.07(2)
C1	C2	1.390(9)		O6	N2	1.048(18)

Table S10. Bond angles for $[Zn(\mathbf{L})_2(NO_3)](NO_3)\cdot H_2O$.

Atom	Atom	Atom	Angle/°		Atom	Atom	Atom	Angle/°
N6	Zn1	N3	173.7(3)		C32	C31	C30	121.3(9)
N6	Zn1	O1	86.1(3)		C13	C18	C17	121.9(9)
N6	Zn1	O2	84.8(2)		C35	C34	C33	123.9(10)
N3	Zn1	O1	88.2(2)		C12	N5	C11	128.1(10)
N3	Zn1	O2	89.8(3)		C12	N5	C7	109.2(10)
O1	Zn1	O2	56.8(2)		C11	N5	C7	122.7(9)
N4	Zn1	N6	104.3(3)		N4	C12	N5	108.6(9)
N4	Zn1	N3	79.0(3)		N4	C12	C13	128.0(10)
N4	Zn1	O1	96.5(2)		N5	C12	C13	123.4(10)
N4	Zn1	O2	151.6(2)		C10	C11	N5	117.3(9)
N4	Zn1	N7	116.8(2)		C9	C8	C7	119.3(9)
N7	Zn1	N6	79.4(4)		C8	C9	C10	120.7(10)
N7	Zn1	N3	104.1(3)		C6	C7	N5	104.5(10)
N7	Zn1	O1	146.0(2)		C6	C7	C8	137.0(11)
N7	Zn1	O2	91.2(2)		N5	C7	C8	118.4(9)
C21	C20	C19	119.2(9)		C17	C16	C15	121.7(11)
C19	N6	Zn1	127.0(10)		C16	C17	C18	118.4(10)
C19	N6	C23	117.6(7)		C18	C13	C12	120.1(13)
C23	N6	Zn1	115.3(9)		C14	C13	C18	118.4(9)
C5	N3	Zn1	114.2(6)		C14	C13	C12	121.5(14)
C5	N3	C1	120.4(7)		C13	C14	C15	119.7(9)
C1	N3	Zn1	125.4(7)		C29	C28	C27	121.5(9)
N1	O1	Zn1	93.9(6)		C28	C29	N8	119.4(9)
N4	C6	C5	116.9(9)		C22	C21	C20	119.1(9)
C7	C6	C5	133.6(10)		C25	C24	C23	133.4(10)
C7	C6	N4	109.6(8)		C25	C24	N7	108.9(8)
O3	N1	O1	119.4(10)		N7	C24	C23	117.6(10)
O3	N1	O2	123.6(11)		C21	C22	C23	119.8(9)
O2	N1	O1	117.0(9)		C27	C26	C25	120.2(8)
N3	C5	C6	115.8(9)		C26	C27	C28	119.6(8)
N3	C5	C4	121.1(8)		N6	C19	C20	122.8(8)
C4	C5	C6	123.1(9)		N6	C23	C24	113.5(12)
C30	N8	C29	130.4(10)		N6	C23	C22	121.5(8)
C30	N8	C25	107.3(9)		C22	C23	C24	125.0(13)
C29	N8	C25	122.3(8)		N8	C25	C26	117.1(9)
C2	C3	C4	118.9(8)		C24	C25	N8	105.9(10)
C5	C4	C3	118.8(8)		C24	C25	C26	137.0(11)
N8	C30	C31	123.5(9)		C30	N7	Zn1	138.1(7)
N7	C30	N8	111.1(9)		C30	N7	C24	106.8(8)
N7	C30	C31	125.3(10)		C24	N7	Zn1	113.3(6)
N1	O2	Zn1	92.3(6)		C31	C32	C33	120.1(9)
C31	C36	C35	121.0(9)		C3	C2	C1	119.9(8)
C6	N4	Zn1	114.0(7)		C34	C33	C32	117.7(9)
C12	N4	Zn1	137.6(8)		C16	C15	C14	119.8(10)

C12	N4	C6	108.1(8)		C11	C10	C9	121.6(10)
N3	C1	C2	121.0(8)		O4	N2	O5	111(3)
C34	C35	C36	117.4(9)		O6	N2	O5	117(4)
C36	C31	C30	118.7(9)		O6	N2	O4	130(5)
C36	C31	C32	119.9(9)					

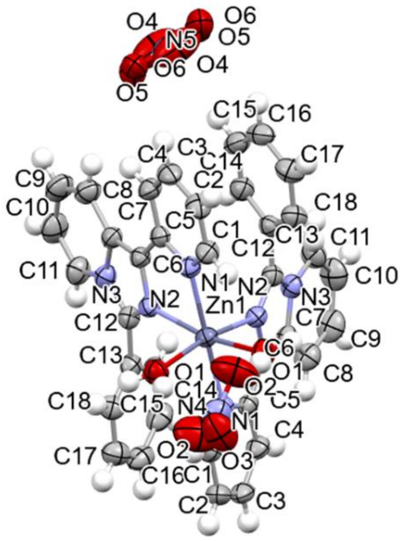

Figure S15. Asymmetric unit of [Zn(**L**)$_2$(H_2O)$_2$](NO_3)$_2$.

Table S11. Crystal data and structure refinement for [Zn(**L**)$_2$(H_2O)$_2$](NO_3)$_2$.

Empirical formula	$C_{36}H_{30}N_8O_8Zn$
Formula weight	768.05
Temperature/K	298.00
Crystal system	Monoclinic
Space group	C2/c
a/Å	18.7794(9)
b/Å	11.4113(5)
c/Å	16.2186(7)
α/°	90
β/°	107.670(5)
γ/°	90
Volume/Å^3	3311.6(3)
Z	4
ρ_{calc}g/cm^3	1.540
μ/mm^{-1}	1.614
F(000)	1584.0
Crystal size/mm^3	0.15 × 0.12 × 0.1
Radiation	CuKα (λ = 1.54184)
2Θ range for data collection/°	9.192 to 135.004
Index ranges	-21 ≤ h ≤ 22, -13 ≤ k ≤ 13, -12 ≤ l ≤ 19
Reflections collected	7167

Independent reflections	2756 [R_{int} = 0.0345, R_{sigma} = 0.0386]
Data/restraints/parameters	2756/0/265
Goodness-of-fit on F^2	1.051
Final R indexes [I>=2σ (I)]	R_1 = 0.0459, wR_2 = 0.1151
Final R indexes [all data]	R_1 = 0.0577, wR_2 = 0.1245
Largest diff. peak/hole / e $Å^{-3}$	0.58/-0.38

Table S12. Bond lengths for $[Zn(\mathbf{L})_2(H_2O)_2](NO_3)_2$.

Atom	Atom	Length/Å		Atom	Atom	Length/Å
Zn1	O1^{1}	2.169(2)		C3	C2	1.375(5)
Zn1	O1	2.169(2)		C2	C1	1.370(4)
Zn1	N1	2.143(2)		C11	C10	1.338(5)
Zn1	N1^{1}	2.143(2)		C14	C15	1.385(5)
Zn1	N2^{1}	2.169(2)		C18	C17	1.379(4)
Zn1	N2	2.169(2)		C10	C9	1.417(5)
N3	C7	1.397(4)		C9	C8	1.353(5)
N3	C12	1.376(4)		C15	C16	1.372(5)
N3	C11	1.385(4)		C17	C16	1.382(5)
C5	N1	1.348(4)		N4	O2^{1}	1.216(4)
C5	C4	1.384(4)		N4	O2	1.216(4)
C5	C6	1.461(4)		N4	O3	1.224(5)
N1	C1	1.334(4)		N5	O6	1.342(6)
C7	C6	1.383(4)		N5	O6^{2}	1.342(6)
C7	C8	1.425(4)		N5	O5^{2}	1.236(8)
N2	C12	1.318(4)		N5	O5	1.236(8)
N2	C6	1.374(3)		N5	O4	1.130(9)
C4	C3	1.381(4)		N5	O4^{2}	1.130(9)
C13	C12	1.462(4)		O6	O5	1.459(12)
C13	C14	1.393(4)		O6	O4	1.258(15)
C13	C18	1.389(4)		O5	O4^{2}	1.044(11)
^{1}1-X,+Y,3/2-Z; ^{2}1/2-X,1/2-Y,2-Z						

Table S13. Bond angles for $[Zn(\mathbf{L})_2(H_2O)_2](NO_3)_2$.

Atom	Atom	Atom	Angle/°		Atom	Atom	Atom	Angle/°
O1	Zn1	O1^{1}	85.28(16)		N2	C6	C5	118.3(2)
O1	Zn1	N2^{1}	96.30(10)		N2	C6	C7	109.1(2)
O1	Zn1	N2	168.24(9)		N1	C1	C2	123.2(3)
O1^{1}	Zn1	N2	96.30(10)		C10	C11	N3	119.2(3)
O1^{1}	Zn1	N2^{1}	168.24(9)		C15	C14	C13	119.9(3)
N1^{1}	Zn1	O1	88.52(9)		C17	C18	C13	120.2(3)
N1	Zn1	O1	91.71(9)		C11	C10	C9	120.2(3)
N1^{1}	Zn1	O1^{1}	91.71(9)		C8	C9	C10	121.4(3)
N1	Zn1	O1^{1}	88.52(9)		C9	C8	C7	119.3(3)
N1^{1}	Zn1	N1	179.68(13)		C16	C15	C14	120.1(3)
N1^{1}	Zn1	N2	103.05(9)		C18	C17	C16	119.9(3)
N1^{1}	Zn1	N2^{1}	76.71(9)		C15	C16	C17	120.5(3)
N1	Zn1	N2^{1}	103.05(9)		O2	N4	O2^{1}	119.4(5)
N1	Zn1	N2	76.71(9)		O2	N4	O3	120.3(3)

N2^{1}	Zn1	N2	84.52(12)		O2^{1}	N4	O3	120.3(3)
C12	N3	C7	108.1(2)		O6	N5	O6^{2}	180.0
C12	N3	C11	129.4(3)		O5	N5	O6	68.8(5)
C11	N3	C7	122.4(3)		O5^{2}	N5	O6^{2}	68.8(5)
N1	C5	C4	120.9(3)		O5	N5	O6^{2}	111.2(5)
N1	C5	C6	114.5(2)		O5^{2}	N5	O6	111.2(5)
C4	C5	C6	124.6(3)		O5^{2}	N5	O5	180.0
C5	N1	Zn1	116.50(18)		O4	N5	O6	60.4(7)
C1	N1	Zn1	124.8(2)		O4^{2}	N5	O6	119.5(7)
C1	N1	C5	118.7(2)		O4	N5	O6^{2}	119.6(7)
N3	C7	C8	117.3(3)		O4^{2}	N5	O6^{2}	60.5(7)
C6	C7	N3	105.0(2)		O4^{2}	N5	O5^{2}	127.9(6)
C6	C7	C8	137.6(3)		O4	N5	O5	127.9(6)
C12	N2	Zn1	137.02(19)		O4^{2}	N5	O5	52.1(6)
C12	N2	C6	108.4(2)		O4	N5	O5^{2}	52.1(6)
C6	N2	Zn1	112.12(18)		O4^{2}	N5	O4	180.0
C3	C4	C5	119.5(3)		N5	O6	O5	52.2(3)
C14	C13	C12	120.5(3)		O4	O6	N5	51.4(4)
C18	C13	C12	120.0(3)		O4	O6	O5	102.7(6)
C18	C13	C14	119.5(3)		N5	O5	O6	59.1(5)
N3	C12	C13	124.7(3)		O4^{2}	O5	N5	58.7(8)
N2	C12	N3	109.2(2)		O4^{2}	O5	O6	116.4(12)
N2	C12	C13	126.1(3)		N5	O4	O6	68.2(8)
C2	C3	C4	119.1(3)		O5^{2}	O4	N5	69.2(8)
C1	C2	C3	118.5(3)		O5^{2}	O4	O6	135.0(13)
C7	C6	C5	132.5(3)					
[1]1-X,+Y,3/2-Z; [2]1/2-X,1/2-Y,2-Z								

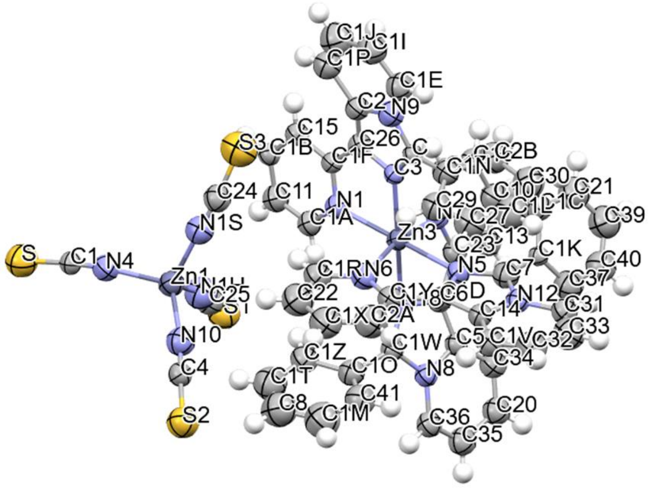

Figure S16. Asymmetric unit of $[Zn(\mathbf{L})_3][Zn(SCN)_4]$.

Table S14. Crystal data and structure refinement for [Zn(**L**)$_3$][Zn(SCN)$_4$].

Empirical formula	$C_{58}H_{39}N_{13}S_4Zn_2$
Formula weight	1177.00
Temperature/K	298.00
Crystal system	Triclinic
Space group	P-1
a/Å	14.003(5)
b/Å	14.721(5)
c/Å	15.586(5)
α/°	72.84(3)
β/°	84.61(3)
γ/°	62.59(4)
Volume/Å^3	2721.9(19)
Z	2
ρ_{calc}g/cm^3	1.436
μ/mm^{-1}	1.087
F(000)	1204.0
Crystal size/mm^3	0.09 × 0.08 × 0.02
Radiation	MoKα (λ = 0.71073)
2Θ range for data collection/°	4.168 to 58.984
Index ranges	-19 ≤ h ≤ 18, -20 ≤ k ≤ 17, -21 ≤ l ≤ 15
Reflections collected	25360
Independent reflections	12805 [R_{int} = 0.4426, R_{sigma} = 1.1851]
Data/restraints/parameters	12805/462/695
Goodness-of-fit on F^2	0.853
Final R indexes [I>=2σ (I)]	R_1 = 0.1414, wR_2 = 0.2906
Final R indexes [all data]	R_1 = 0.5490, wR_2 = 0.6456
Largest diff. peak/hole / e Å^{-3}	1.09/-0.81

Table S15. Bond lengths for [Zn(**L**)$_3$][Zn(SCN)$_4$].

Atom	Atom	Length/Å		Atom	Atom	Length/Å
Zn3	N1	2.157(17)		C16	C17	1.36(3)
Zn3	C3	2.166(16)		N18	C1W	1.30(2)
Zn3	N5	2.231(17)		N18	C6	1.39(2)
Zn3	N6	2.12(2)		C1C	C1K	1.38(3)
Zn3	N7	2.160(17)		C1C	C21	1.44(3)
Zn3	N18	2.326(18)		C36	C35	1.32(3)
Zn1	N1H	1.99(2)		C1D	C1Y	1.51(3)
Zn1	N1S	1.99(2)		C1E	C1I	1.35(3)
Zn1	N4	1.958(18)		C37	C40	1.34(3)
Zn1	N10	1.98(2)		C37	C1K	1.41(3)
S2	C4	1.63(3)		C1F	C26	1.44(3)

S1	C25	1.59(3)		C40	C39	1.30(4)
S	C1	1.59(2)		N1H	C25	1.16(3)
S3	C24	1.62(3)		C35	C20	1.41(3)
N1	C1A	1.35(2)		C1I	C1J	1.46(3)
N1	C1F	1.32(2)		C1J	C1P	1.37(3)
C2	N9	1.46(3)		C1K	C7	1.42(3)
C2	C1P	1.52(3)		C1L	C27	1.26(3)
C2	C26	1.30(3)		C1L	C30	1.36(4)
C3	C	1.32(2)		C31	C33	1.35(3)
C3	C26	1.36(2)		C1M	C41	1.47(4)
C	N9	1.35(2)		C1M	C8	1.37(4)
C	C1N	1.50(3)		C34	C20	1.34(3)
N5	C1D	1.47(3)		C34	C5	1.46(3)
N5	C7	1.37(2)		C1N	C29	1.38(3)
N6	C1R	1.37(3)		C1N	C2B	1.44(3)
N6	C1Y	1.36(3)		C32	C1V	1.26(3)
N7	C17	1.39(3)		C32	C33	1.36(3)
N7	C23	1.38(2)		C1O	C1W	1.59(3)
N8	C36	1.42(3)		C1O	C1Z	1.48(3)
N8	C1W	1.39(2)		C1O	C41	1.27(3)
N8	C5	1.37(2)		C1R	C22	1.41(3)
N9	C1E	1.37(3)		N1S	C24	1.11(3)
C10	C13	1.36(3)		C1T	C1Z	1.41(4)
C10	C16	1.40(3)		C1T	C8	1.24(4)
C11	C1A	1.38(3)		C1X	C22	1.35(4)
C11	C1B	1.36(3)		C1X	C2A	1.33(3)
N12	C14	1.36(2)		C1Y	C2A	1.36(3)
N12	C31	1.44(2)		C21	C39	1.38(3)
N12	C7	1.42(3)		C27	C29	1.36(3)
C13	C23	1.40(3)		C2B	C30	1.58(4)
C14	C1D	1.39(3)		C23	C6	1.52(3)
C14	C1V	1.46(3)		N4	C1	1.19(2)
C15	C1B	1.36(2)		C4	N10	1.11(3)
C15	C1F	1.44(3)		C6	C5	1.37(3)

Table S16. Bond angles for [Zn(**L**)$_3$][Zn(SCN)$_4$].

Atom	Atom	Atom	Angle/°		Atom	Atom	Atom	Angle/°
N1	Zn3	C3	76.4(6)		C1I	C1E	N9	115(2)
N1	Zn3	N5	174.3(6)		C40	C37	C1K	119(3)
N1	Zn3	N7	90.2(6)		N1	C1F	C15	120.1(17)
N1	Zn3	N18	99.9(6)		N1	C1F	C26	118.4(19)

C3	Zn3	N5	99.9(6)		C26	C1F	C15	121.2(19)
C3	Zn3	N18	174.3(7)		C39	C40	C37	126(3)
N5	Zn3	N18	84.1(6)		C25	N1H	Zn1	166(2)
N6	Zn3	N1	96.9(7)		C36	C35	C20	119(3)
N6	Zn3	C3	92.5(7)		C1E	C1I	C1J	120(3)
N6	Zn3	N5	78.8(7)		C1P	C1J	C1I	127(3)
N6	Zn3	N7	166.9(7)		C1C	C1K	C37	121(2)
N6	Zn3	N18	92.3(7)		C1C	C1K	C7	119(2)
N7	Zn3	C3	99.9(6)		C37	C1K	C7	120(2)
N7	Zn3	N5	94.8(6)		C27	C1L	C30	122(3)
N7	Zn3	N18	75.6(6)		C33	C31	N12	117(2)
N1H	Zn1	N1S	111.3(9)		C8	C1M	C41	112(3)
N4	Zn1	N1H	107.2(8)		C20	C34	C5	118(2)
N4	Zn1	N1S	106.9(8)		C29	C1N	C	123(2)
N4	Zn1	N10	114.2(8)		C29	C1N	C2B	123(2)
N10	Zn1	N1H	107.3(9)		C2B	C1N	C	114(2)
N10	Zn1	N1S	109.9(8)		C1V	C32	C33	123(3)
C1A	N1	Zn3	126.1(15)		C1Z	C1O	C1W	114(2)
C1F	N1	Zn3	113.7(13)		C41	C1O	C1W	121(2)
C1F	N1	C1A	118.6(19)		C41	C1O	C1Z	125(2)
N9	C2	C1P	112.1(19)		C1J	C1P	C2	115(2)
C26	C2	N9	108(2)		N6	C1R	C22	122(3)
C26	C2	C1P	140(2)		C24	N1S	Zn1	172(2)
C	C3	Zn3	138.8(14)		C8	C1T	C1Z	123(3)
C	C3	C26	107.0(16)		C32	C1V	C14	120(2)
C26	C3	Zn3	113.8(12)		N8	C1W	C1O	123(2)
C3	C	N9	112.1(17)		N18	C1W	N8	109.6(18)
C3	C	C1N	130.3(18)		N18	C1W	C1O	127(2)
N9	C	C1N	116.9(17)		C2A	C1X	C22	118(3)
C1D	N5	Zn3	108.5(12)		N6	C1Y	C1D	112(2)
C7	N5	Zn3	141.5(16)		N6	C1Y	C2A	124(2)
C7	N5	C1D	109.9(18)		C2A	C1Y	C1D	124(2)
C1R	N6	Zn3	125.6(18)		C1T	C1Z	C1O	111(3)
C1Y	N6	Zn3	120.3(15)		C34	C20	C35	124(3)
C1Y	N6	C1R	114(2)		C39	C21	C1C	125(3)
C17	N7	Zn3	122.3(14)		C1X	C22	C1R	120(3)
C23	N7	Zn3	117.6(15)		N1S	C24	S3	174(3)
C23	N7	C17	120.1(19)		N1H	C25	S1	177(2)
C1W	N8	C36	127.3(19)		C1L	C27	C29	125(3)
C5	N8	C36	125.1(19)		C27	C29	C1N	120(3)
C5	N8	C1W	107.6(18)		C1X	C2A	C1Y	122(3)
C	N9	C2	102.8(17)		C1N	C2B	C30	111(2)

C	N9	C1E	126(2)		N7	C23	C13	123(2)
C1E	N9	C2	131(2)		N7	C23	C6	113.4(19)
C13	C10	C16	126(2)		C13	C23	C6	123(2)
C1B	C11	C1A	116(2)		C2	C26	C3	110.5(19)
C14	N12	C31	121(2)		C2	C26	C1F	134(2)
C14	N12	C7	114.5(18)		C3	C26	C1F	115.9(19)
C7	N12	C31	124.9(19)		C1L	C30	C2B	120(3)
C10	C13	C23	113(2)		C31	C33	C32	122(3)
N12	C14	C1D	106(2)		C40	C39	C21	115(3)
N12	C14	C1V	116.9(19)		C1	N4	Zn1	158.6(17)
C1D	C14	C1V	137(2)		N4	C1	S	176(2)
C1B	C15	C1F	118(2)		N10	C4	S2	176(2)
C17	C16	C10	119(3)		C4	N10	Zn1	172(2)
C16	C17	N7	118(2)		N18	C6	C23	118.2(19)
C1W	N18	Zn3	137.3(15)		C5	C6	N18	109(2)
C1W	N18	C6	107.9(18)		C5	C6	C23	133(2)
C6	N18	Zn3	106.8(14)		N8	C5	C34	116(2)
N1	C1A	C11	125(2)		C6	C5	N8	106.1(19)
C11	C1B	C15	123(2)		C6	C5	C34	138(2)
C1K	C1C	C21	113(2)		N5	C7	N12	103(2)
C35	C36	N8	117(2)		N5	C7	C1K	131(2)
N5	C1D	C1Y	119.8(19)		N12	C7	C1K	125.5(19)
C14	C1D	N5	106.7(17)		C1O	C41	C1M	121(3)
C14	C1D	C1Y	133(2)		C1T	C8	C1M	129(4)

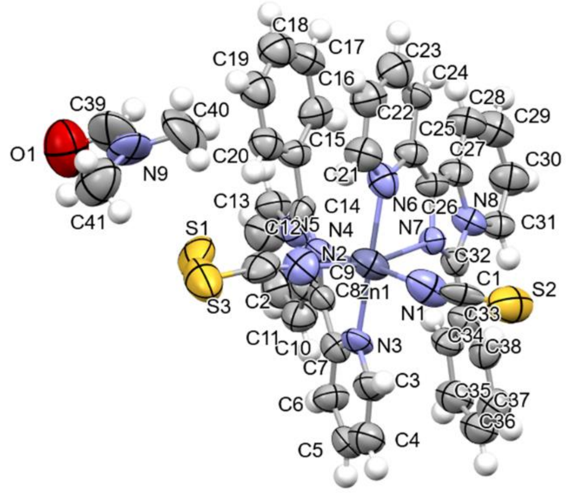

Figure S17. Asymmetric unit of [Zn(**L**)$_2$(SCN)$_2$]·DMF.

Table S17. Crystal data and structure refinement for [Zn(**L**)$_2$(SCN)$_2$]·DMF.

Empirical formula	$C_{41}H_{33}N_9OS_2Zn$	
Formula weight	797.25	

Temperature/K	298.00	
Crystal system	triclinic	
Space group	P-1	
a/Å	11.077(3)	
b/Å	11.531(4)	
c/Å	15.823(3)	
α/°	94.80(2)	
β/°	107.74(2)	
γ/°	95.65(3)	
Volume/$Å^3$	1901.8(10)	
Z	2	
ρ_{calc}g/cm^3	1.392	
μ/mm^{-1}	2.292	
F(000)	824.0	
Crystal size/mm^3	0.11 × 0.1 × 0.08	
Radiation	CuKα (λ = 1.54184)	
2Θ range for data collection/°	7.76 to 135.944	
Index ranges	-9 ≤ h ≤ 13, -8 ≤ k ≤ 13, -12 ≤ l ≤ 19	
Reflections collected	6929	
Independent reflections	6929 [R_{int} = 0.0864, R_{sigma} = 0.1382]	
Data/restraints/parameters	6929/246/498	
Goodness-of-fit on F^2	1.027	
Final R indexes [I>=2σ (I)]	R_1 = 0.0653, wR_2 = 0.1355	
Final R indexes [all data]	R_1 = 0.1247, wR_2 = 0.1820	
Largest diff. peak/hole / e $Å^{-3}$	0.24/-0.39	

Table S18. Bond lengths for [Zn(**L**)$_2$(SCN)$_2$]·DMF.

Atom	Atom	Length/Å		Atom	Atom	Length/Å
Zn1	N7	2.285(10)		C35	C36	1.372(16)
Zn1	N4	2.312(11)		C6	C7	1.391(15)
Zn1	N1	2.070(12)		C36	C37	1.381(15)
Zn1	N3	2.108(10)		C7	C8	1.435(16)
Zn1	N2	2.098(13)		C16	C17	1.402(15)
Zn1	N6	2.118(11)		C16	C15	1.374(15)
S2	C1	1.618(16)		C14	C15	1.458(16)
S3	C2	1.68(2)		C9	C8	1.389(15)
N7	C32	1.325(13)		C9	C10	1.435(15)
N7	C26	1.391(13)		C20	C15	1.370(15)
N5	C14	1.409(13)		C20	C19	1.409(16)
N5	C9	1.383(14)		C11	C12	1.412(17)
N5	C13	1.374(13)		C11	C10	1.340(15)
N4	C14	1.323(13)		C12	C13	1.369(15)
N4	C8	1.371(14)		C24	C25	1.427(15)
N1	C1	1.145(15)		C24	C23	1.391(15)
N3	C3	1.323(13)		C25	N6	1.366(14)
N3	C7	1.365(14)		N6	C21	1.331(14)
N2	C2	1.101(17)		N8	C31	1.405(14)
C34	C33	1.411(14)		C31	C30	1.346(14)
C34	C35	1.395(15)		C30	C29	1.404(16)
C38	C33	1.380(15)		C28	C29	1.374(16)
C38	C37	1.393(15)		C21	C22	1.392(16)
C32	C33	1.486(16)		C23	C22	1.360(16)
C32	N8	1.384(14)		C17	C18	1.349(16)
C26	C27	1.414(16)		C18	C19	1.343(17)
C26	C25	1.411(15)		C39	O1	1.28(2)
C27	N8	1.368(14)		C39	N9	1.32(2)
C27	C28	1.448(15)		C41	N9	1.397(17)
C3	C4	1.388(15)		C40	N9	1.495(19)
C5	C6	1.360(14)		C2	S1	1.63(4)
C5	C4	1.382(15)				

Table S19. Bond angles for [Zn(**L**)$_2$(SCN)$_2$]·DMF.

Atom	Atom	Atom	Angle/°		Atom	Atom	Atom	Angle/°
N7	Zn1	N4	76.4(3)		N3	C7	C8	114.1(14)
N1	Zn1	N7	93.7(5)		C6	C7	C8	124.6(15)
N1	Zn1	N4	164.7(5)		C5	C4	C3	115.8(14)
N1	Zn1	N3	95.1(5)		C15	C16	C17	119.2(15)
N1	Zn1	N2	99.7(6)		N5	C14	C15	122.1(14)

N1	Zn1	N6	94.8(4)		N4	C14	N5	106.5(13)
N3	Zn1	N7	94.2(4)		N4	C14	C15	131.4(14)
N3	Zn1	N4	74.3(5)		N5	C9	C8	104.5(13)
N3	Zn1	N6	166.0(5)		N5	C9	C10	119.2(14)
N2	Zn1	N7	163.5(5)		C8	C9	C10	136.3(16)
N2	Zn1	N4	92.2(5)		C15	C20	C19	117.8(15)
N2	Zn1	N3	94.2(5)		N4	C8	C7	119.0(14)
N2	Zn1	N6	93.9(5)		N4	C8	C9	109.3(14)
N6	Zn1	N7	75.3(5)		C9	C8	C7	131.5(16)
N6	Zn1	N4	93.9(4)		C10	C11	C12	123.8(15)
C32	N7	Zn1	138.3(10)		C13	C12	C11	117.8(15)
C32	N7	C26	108.6(12)		C12	C13	N5	120.5(14)
C26	N7	Zn1	108.5(9)		C11	C10	C9	117.2(15)
C9	N5	C14	109.7(12)		C23	C24	C25	119.1(14)
C13	N5	C14	128.6(14)		C26	C25	C24	124.7(15)
C13	N5	C9	121.1(13)		N6	C25	C26	116.1(13)
C14	N4	Zn1	137.4(10)		N6	C25	C24	119.2(13)
C14	N4	C8	110.0(12)		C25	N6	Zn1	117.5(10)
C8	N4	Zn1	108.8(10)		C21	N6	Zn1	122.3(11)
C1	N1	Zn1	170.8(13)		C21	N6	C25	120.1(13)
C3	N3	Zn1	122.9(11)		C32	N8	C31	128.2(14)
C3	N3	C7	117.3(13)		C27	N8	C32	109.0(13)
C7	N3	Zn1	119.8(10)		C27	N8	C31	122.5(13)
C2	N2	Zn1	168(2)		C30	C31	N8	117.6(13)
C35	C34	C33	119.1(13)		C31	C30	C29	120.4(15)
C33	C38	C37	118.8(14)		C29	C28	C27	113.9(14)
N7	C32	C33	127.0(14)		C28	C29	C30	124.7(15)
N7	C32	N8	109.1(13)		N6	C21	C22	122.4(14)
N8	C32	C33	123.9(14)		C22	C23	C24	119.9(15)
N7	C26	C27	107.5(13)		C23	C22	C21	119.2(16)
N7	C26	C25	118.8(13)		N1	C1	S2	178.6(16)
C25	C26	C27	133.7(15)		C18	C17	C16	119.3(15)
C26	C27	C28	133.3(15)		C16	C15	C14	123.5(15)
N8	C27	C26	105.9(13)		C20	C15	C16	121.3(15)
N8	C27	C28	120.8(14)		C20	C15	C14	115.1(15)
C34	C33	C32	119.5(13)		C36	C37	C38	120.1(14)
C38	C33	C34	120.9(14)		C19	C18	C17	121.7(16)
C38	C33	C32	119.5(13)		C18	C19	C20	120.7(16)
N3	C3	C4	125.3(14)		O1	C39	N9	118(2)
C6	C5	C4	121.2(15)		C39	N9	C41	122.7(19)
C36	C35	C34	119.2(14)		C39	N9	C40	119.1(19)
C5	C6	C7	119.1(15)		C41	N9	C40	118.2(17)

C35	C36	C37	121.7(15)		N2	C2	S3	168(2)
N3	C7	C6	121.2(14)		N2	C2	S1	151(2)

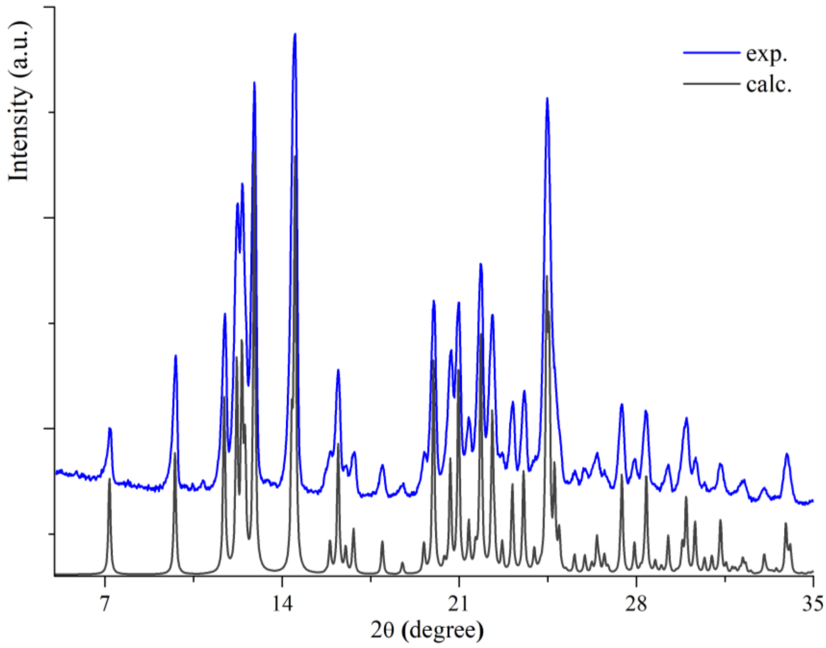

Figure S18. Experimental and calculated PXRD pattern of [Zn(**L**)$(NO_3)_2$].

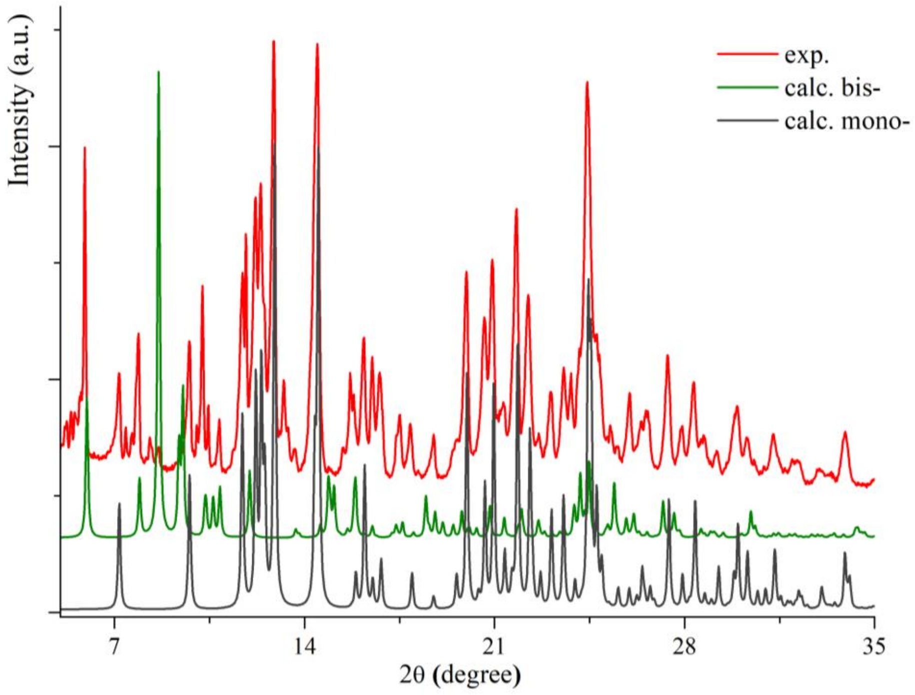

Figure S19. Experimental pattern of direct reaction between $Zn(NO_3)_2$ and **L**, and calculated PXRD patterns of [Zn(**L**)$_2$(NO_3)](NO_3)·H_2O and [Zn(**L**)(NO_3)$_2$].

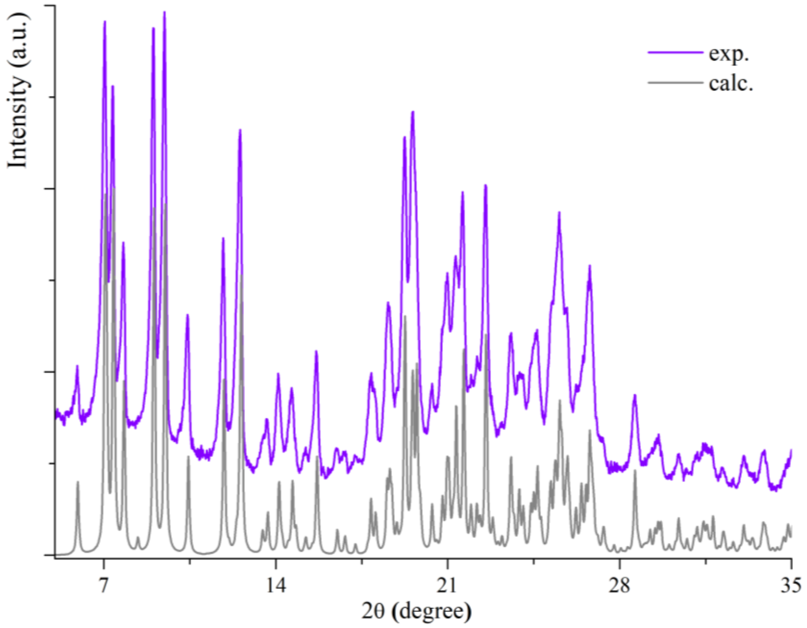

Figure S20. Experimental and calculated PXRD pattern of [Zn(**L**)$_3$][Zn(SCN)$_4$].
